# Identification and characterization of SSR, SNP and InDel molecular markers from RNA-Seq data of guar (*Cyamopsis tetragonoloba*, L. Taub.) roots

**DOI:** 10.1186/s12864-018-5205-9

**Published:** 2018-12-20

**Authors:** Omika Thakur, Gursharn Singh Randhawa

**Affiliations:** 1 Department of Biotechnology, Indian Institute of Technology Roorkee, -247667, Roorkee, Uttarakhand India; 2 Present address: Department of Biochemistry & Biotechnology, Sardar Bhagwan Singh University, Balawala, Dehradun, Uttarakhand 248161 India

**Keywords:** RNA-Seq, Molecular markers, Simple sequence repeats, Single nucleotide polymorphisms, Insertions and deletions, Marker-assisted selection

## Abstract

**Background:**

Guar [*Cyamopsis tetragonoloba*, L. Taub.] is an important industrial crop because of the commercial applications of the galactomannan gum contained in its seeds. Plant breeding programmes based on marker-assisted selection require a rich resource of molecular markers. As limited numbers of such markers are available for guar, molecular breeding programmes have not been undertaken for the genetic improvement of this important crop. Hence, the present work was done to enrich the molecular markers resource of guar by identifying high quality SSR, SNP and InDel markers from the RNA-Seq data of the roots of two guar varieties.

**Results:**

We carried out RNA-Seq analysis of the roots of two guar varieties, namely, RGC-1066 and M-83. A total of 102,479 unigenes with an average length of 1016 bp were assembled from about 30 million high quality pair-end reads generated by an Illumina HiSeq 2500 platform. The assembled unigenes had 86.55% complete and 97.71% partially conserved eukaryotic genes (CEGs). The functional annotation of assembled unigenes using BLASTX against six databases showed that the guar unigenes were most similar to *Glycine max*. We could assign GO terms to 45,200 unigenes using the UniProt database. The screening of 102,479 unigenes with MISA and SAMtools version 1.4 softwares resulted in the identification of 25,040 high-confidence molecular markers which consisted of 18,792 SSRs, 5999 SNPs and 249 InDels. These markers tagged most of the genes involved in root development, stress tolerance and other general metabolic activities. Each of the 25,040 molecular markers was characterized, particularly with respect to its position in the unigene. For 71% of the molecular markers, we could determine the names, products and functions of the unigenes. About 80% of the markers, from a random sample of molecular markers, showed PCR amplification.

**Conclusions:**

We have identified and characterized 25,040 high confidence SSR, SNP and InDel molecular markers in guar. It is expected that these markers will be useful in molecular breeding programmes and will also be helpful in studying molecular mechanisms of root development, stress tolerance and gum synthesis in guar.

**Electronic supplementary material:**

The online version of this article (10.1186/s12864-018-5205-9) contains supplementary material, which is available to authorized users.

## Background

Guar [*Cyamopsis tetragonoloba*, L. Taub.] is a diploid, annual and drought-tolerant leguminous crop mainly cultivated in the semi-arid areas of India, Pakistan and the United States of America. It is generally grown in marginal soils having nitrogen and water deficiencies and often containing high salt concentrations. Traditionally grown for fodder, green manure and vegetable purposes, guar has recently been recognized as an important industrial crop because of the presence of gum in its seeds [1, 2]. The guar gum, which is mainly galactomannan, is a natural thickener used in petroleum, food, paper, textile, cosmetics and pharmaceutical industries [3]. It has also shown a potential in the treatment of diseases like irritable bowel syndrome, diarrhea, high cholesterol and diabetes [4–6]. Due to the high demand of guar gum all over the world, improved varieties of guar, having increased amounts of high quality gum and also able to grow under adverse conditions, have become a necessity.

The plant breeding programmes based on marker-assisted selection require the availability of a large number of molecular markers for gene tagging, genetic mapping and map-based cloning of important genes [7, 8]. Six molecular markers, namely, random amplified polymorphic DNA (RAPD), ribosomal DNA (rDNA), inter simple sequence repeat (ISSR), sequence characterized amplified region (SCAR), simple sequence repeat (SSR) and single nucleotide polymorphisms (SNPs) have been identified in guar [9–17]. The last two markers, namely, SSRs and SNPs, are considered very important for genetic mapping and plant breeding programmes [18]. Insertions and deletions (InDels) have been also found to be very valuable markers in the plant breeding programmes [19]. As the guar genome has not been sequenced, fewer SSR and SNP markers, are available for guar as compared to those available in other well-studied legume crops like soybean, *Lotus, Medicago*, pigeonpea and chickpea [20–25]. Therefore, further research is needed to generate a large number of SSR, SNP and InDel markers for guar.

The sequencing of cDNA pools obtained from RNA samples from various tissues, using the next generation sequencing (NSG) technique, provides a large collection of expressed sequences. This approach, called RNA-Seq technology, can be used for generating SSR and SNP molecular markers [26, 27]. The molecular markers, thus generated, are likely to show more transferability than the other markers because of their presence in the genomic regions that are more conserved [28]. The RNA-Seq approach has been successfully used to generate SSR and SNP molecular markers in guar [16, 17]. However, the number of the markers obtained is still far less than those available in the other legumes, for which the complete genomic sequences are available. Moreover, there is only one report of SNP identification in guar [16] and in the report the characterization of identified SNP markers has not been done. The SNP markers need thorough characterization for their appropriate and efficient use. The breeding programmes for obtaining improved varieties of guar had limited success so far because of the shortage of useful molecular markers. Hence, the present work was started with the objective of identifying and characterizing SSR, SNP and InDel markers from the root tissue of guar to enrich the molecular marker resource of this important industrial crop. The root tissue was selected as the root specific processes are considered of particular importance in the abiotic stress responses of plants [29]. The two varieties, namely, RGC-1066 and M-83 having contrasting characters were selected for this work as these were expected to show significant genetic differences.

## Results

### RNA seq and De novo transcriptome assembly of guar root

**Fig. 1:**
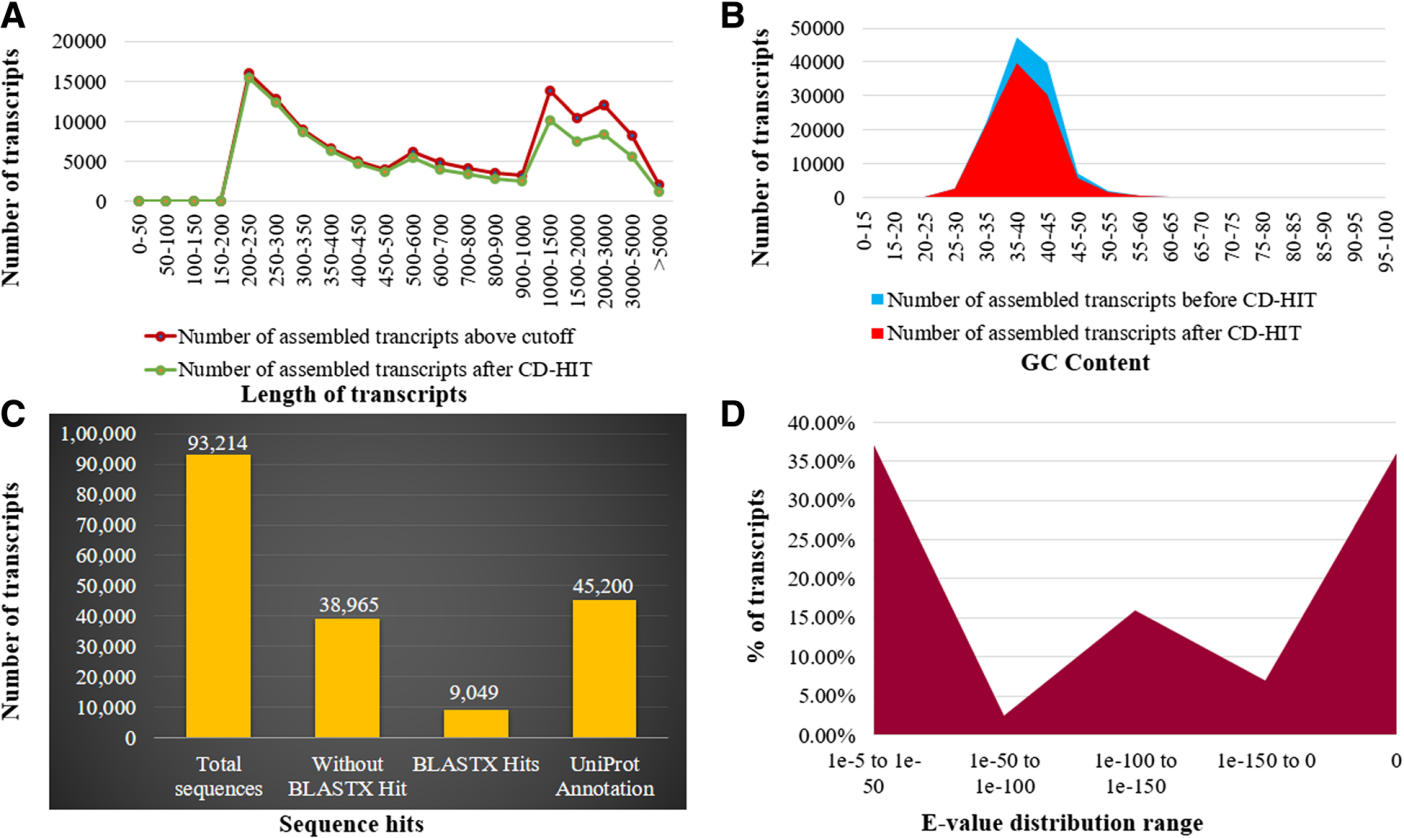
Functional annotation of guar root transcriptome **a**. Length distribution **b**. GC distribution **c**. Distribution of total sequences obtained from Blast2GO three step processes including BLASTX, mapping and annotation of guar root transcriptome **d**. BLASTX E-value distribution

The RNA-Seq of roots of guar varieties RGC-1066 and M-83 by the Illumina HiSeq 2500 sequencing platform generated 29,623,208 and 29,853,028 raw pair-end reads, respectively. The data summary of the guar root transcriptome assembly has been presented in Table 1. The % Q > 30 and % GC for RGC-1066 and M-83 varieties were about 87 and about 44, respectively. The numbers of clean reads generated after trimming adaptors and removing low quality bases were 17,305,480 and 17,517,086 in guar varieties RGC-1066 and M-83, respectively. The % Q > 30 and % GC of clean reads of RGC-1066 and M-83 varieties were 97 and 44, respectively (Table 1). The de novo assembly of clean reads by Trinity program generated 1,22,206 contigs. The mean % GC of unigenes was 40 and the longest unigene was 16,844 bp. The clustering of assembled contigs using CD-HIT version 4.5.4 generated 1,02479 unigenes, with mean % GC of 39.82 and longest unigene 16,844 bp (Additional file 1: Table S1). The sequence length and GC content distributions of assembled unigenes before and after CD-HIT are shown in Fig 1a, b. The lengths of 51,229 unigenes were < 500 bp whereas 51,250 unigenes had the lengths more than 500 bp. A total of 32,949 unigenes had over 1000 bp and 1297 unigenes over 5000 bp lengths. The average length and N50 value of the unigenes were 1016.62 bp and 1907 bp, respectively (Table 2). The unigenes assembly had 86.55% complete and 97.71% partial conserved eukaryotic genes (CEGs) against the 248 CEGs as reference (Additional file 2: Table S2).

**Table 1:** The data summary of de novo transcriptome assembly of guar root

Sample name	RGC-1066	M-83
No. of raw reads	29,623,208	29,853,028
No. of bases/Mb	2962.32	2985.3
GC %	44.89	44.435
Q_30	86.755	87.67
Read length	100 X 2	100 X 2
No. of clean reads	17,305,480	17,517,086
No. of bases/Mb	1612.14	1637.48
GC %	44.27	43.625
Q_30	97.345	97.51
Read length	100 X 2	100 X 2

**Table 2:** Statistics of de novo assembly of root transcriptome of guar

Characteristic	Details
Total number of transcripts	1,02479
Min length	201
Max length	16,844
Average length	1016.62
Standard deviation	1150.28
Median length	500
Number of contigs < 500 bp	51,229
Number of contigs ≥500 bp	51,250
Number of contigs ≥1000 bp	32,949
Number of contigs ≥2000 bp	15,360
Number of contigs ≥5000 bp	1297
N50	1907
Contigs in N50	16,574
GC content	39.82%

### Functional annotation of guar root transcriptome

**Fig. 2:**
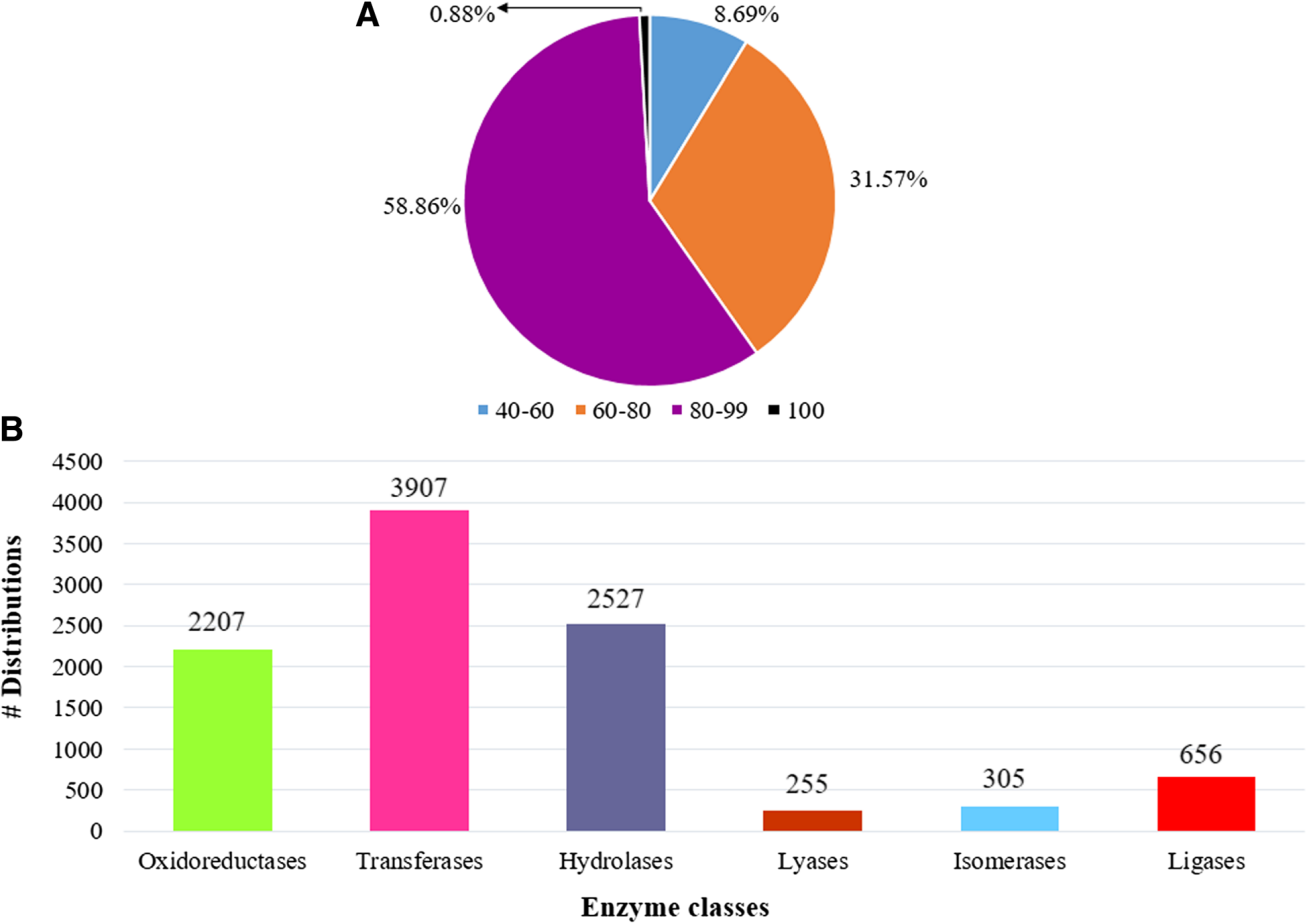
Similarity distributions and enzyme code distributions of assembled unigenes of root transcriptome of guar varieties RGC-1066 and M-83 **a**. Similarity distributions of the top BLAST hits for each sequence against the Nr database **b**. Enzyme code distributions

**Fig. 3:**
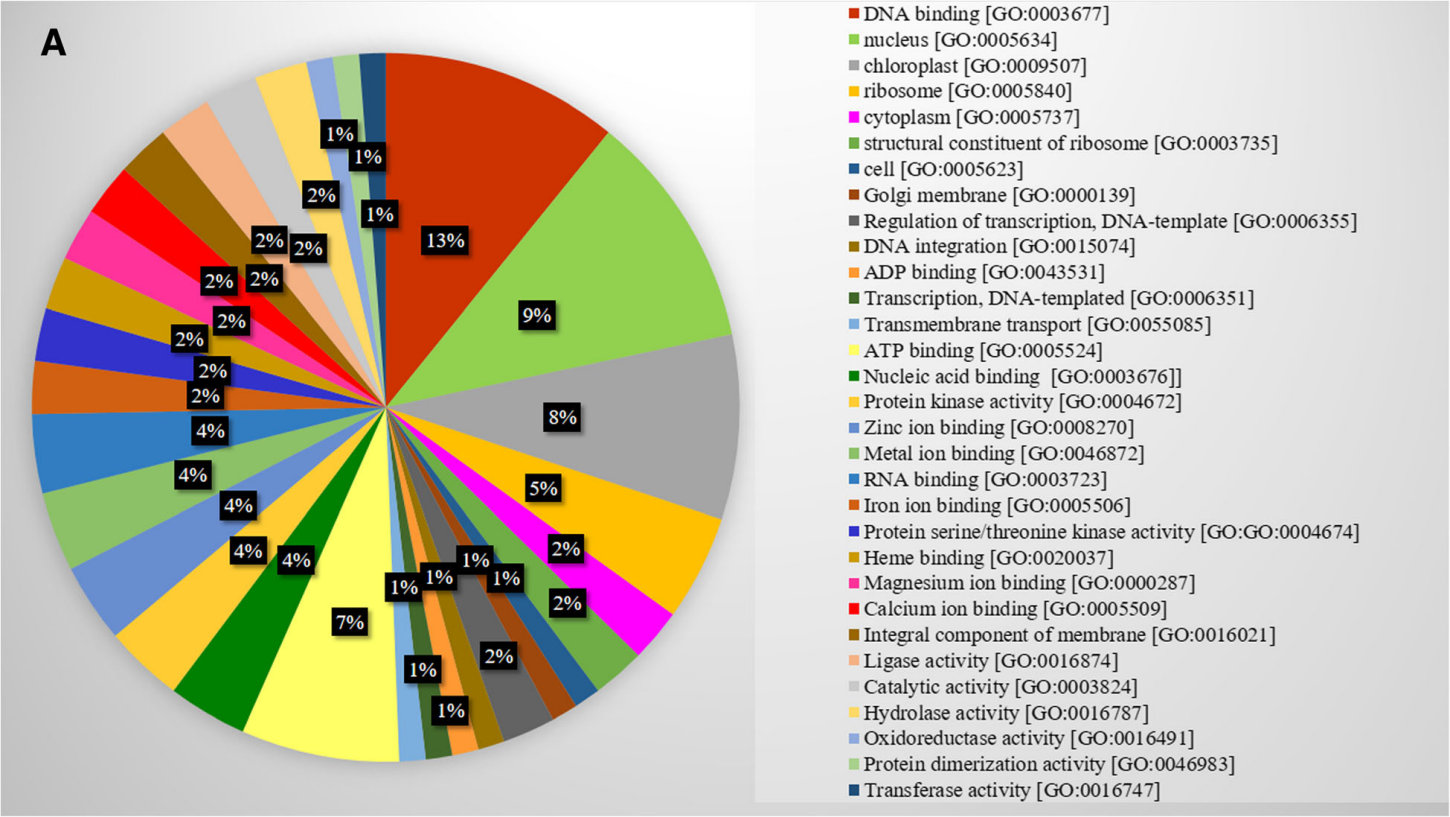
Distribution of biological, molecular and cellular component categories in unigenes of guar root transcriptome

The functional annotation of assembled unigenes of guar root was done using BLASTX against NCBI, UniProt, Pathway, Nt, Pfam and Uniref90 databases, with an E-value cutoff 1e^− 5^ and % identity cutoff 40. A total of 54,249 unigenes were found to have at least one significant hit in UniProt and NCBI databases (Fig 1c). For the remaining 38,965 unigenes, no BLASTX hits were recorded. The top BLASTX hits of unigenes showed that 45.65% guar unigenes had maximum similarity with *Glycine max,* 32.79% with *Cajanus cajan,*15.28% with *Cicer arietinum*, 15.06% with *Glycine soja,*11.86% with *Phaseolus vulgaris*, 10.53% with *Medicago truncatula* and remaining with other legume plants (Additional file 3: Figure S1). About 91.31% of the assembled unigenes had similarity of more than 60% at protein level with the existing proteins in NCBI database. The E-value distribution of 37.32% sequences ranged from 1e^− 50^ to 1e^− 6^ (Fig 1d). The similarity distribution analysis indicated that 59.74% sequences had similarity more than 80 and 40.26% sequences more than 40% with the sequences in NCBI (Fig 2a). Out of the total 102,479 unigenes, we could annotate and assign GO terms to 45,200 unigenes using the UniProt database (Fig 1c). The GO terms were distributed into 60 functional groups which were classified into three categories, namely, cellular components (2106), molecular functions (3889) and biological processes (3392). The top GO terms were ATP binding (2096), nucleus (1046), nucleic acid binding (1045), protein kinase activity (781), ADP binding (774), zinc ion binding (726), metal ion binding (717) and RNA binding (704) (Fig 3; Additional file 4: Table S3). A total of 7426 unigenes were grouped into six enzyme codes: Oxidoreductases (2207), Transferases (3907), Hydrolases (2527), Lyases (255), Isomerases (305) and Ligases (656) (Fig 2b). The most frequent terms among the unigenes annotated by KAAS were “metabolic pathways (905)”, “biosynthesis of secondary metabolities (407)”, “biosynthesis of antibiotics (211)”, “microbial metabolism in diverse environments (157)” and “ribosome (130)” (Additional file 5: Table S4).

### Identification and characterization of 18,792 SSR markers

In order to reduce the bias of transcript length and noise at low level of expression, the unigenes having fragments per kilobase of transcript per million mapped reads (FPKM) ≥1 were selected to identify simple sequence repeats (SSRs) [30]. A total of 18,792 simple sequence repeats (SSRs) belonging to 8 classes of microsatellites were identified in 93,402 unigenes having FPKM ≥1. The characteristics of all the identified SSRs, namely, length of SSR, type of SSR, size of SSR and start and end positions of SSR, have been given in Additional file 6: Table S5. Out of the total 18,792 SSRs, 9983 (52.62%) SSRs were mono-nucleotide, 3455 (18.21%) di-nucleotide, 3914 (20.63%) tri-nucleotide, 373 (1.97%) tetra-nucleotide, 70 (0.37%) penta-nucleotide, 58 (0.31%) hexa-nucleotide, 1073 (5.7%) c1 and 46 (0.25%) c2 motifs. The ‘c1’ are compound motifs containing two types of repeats separated by few nucleotides and c2 are compound motifs in which two types of repeats are not separated by a nucleotide stretch. For each identified SSR, an attempt was made to find the name and characteristics (function/protein product) of the gene containing the SSR. We succeeded in doing so for 12,600 SSRs. The detailed data for mono-, di-, tri-, tetra-, penta-, hexa-, c and c* SSRs have been presented in Additional file 7: Table S6, Additional file 8: Table S7, Additional file 9: Table S8, Additional file 10: Table S9, Additional file 11: Table S10, Additional file 12: Table S11, Additional file 13: Table S12, Additional file 14: Table S13.

For 1732 dinucleotide SSRs, the repeat number varied from 6 to 9, while the repeat number for 2050 trinucleotides, 201 tetranucleotides, 67 pentanucleotides and 50 hexanucleotides were ≤ 5 (Table 3). On an average, the unigenes contained 1 SSR per 5.45 kb. A total of 1073 (5.66%) SSRs were repeated more than 10 times whereas 46 (0.24%) SSRs were repeated more than 20 times. The most abundant class of repeat motifs were mononucleotides (52.62%) followed by tri-nucleotides (20.63%) and dinucleotides (18.21%). The distribution frequencies and per cent distribution of SSRs have been given in Table 4 and Additional file 15: Table S14. The motifs AG, AGG, AAAG, AGAAAG, CT, TTC, CTTT, ATGAG and TGCTAT were more prevalent than the other motifs in the root transcriptome of guar (Additional file 7: Table S6, Additional file 8: Table S7, Additional file 9: Table S8, Additional file 10: Table S9, Additional file 11: Table S10, Additional file 12: Table S11, Additional file 13: Table S12, Additional file 14: Table S13). Out of 20 randomly chosen SSRs, 15 showed amplification in both guar varieties, 3 in RGC-1066 variety and 2 in M-83 variety. The results of amplification of 7 SSRs in both guar varieties have been shown in Additional file 16: Figure S2A.

**Table 3:** Repeat numbers of different SSRs obtained from the guar root transcriptome

Repeat type	Repeat numbers			Total number of repeats
	≤5	6 to 9	≥10	
Mononucleotides	–	–	9983	9983
Dinucleotides	–	1732	1723	3455
Trinucleotides	2050	1334	530	3914
Tetranucleotides	201	172	–	373
Pentanucleotides	67	3	–	70
Hexanucleotides	50	8	–	58

**Table 4:** Percentage distribution frequencies of SSR motif repeats in guar root transcriptome

Repeat number	Repeat type							
	Mono-	Di-	Tri-	Tetra-	Penta-	Hexa-	c	c*
1	0	0	0	0	0	0	0	0
2	0	0.01	0	0	0	0	0	0
3	0	0	0	0	0	0	0	0
4	0	0	0	0	0	0	0	0
5	0	0	11	1	0.3	0.2	0	0
6	0	5	5	0.4	0.01	0.01	0	0
7	0	3	2	0.1	0	0	2	0
8	0	1	2	0.01	0	0	1	0
9	0	1	1	0	0	0	0.1	0
10	14	0.4	0.1	0	0	0	0	0
11	7	0.4	0.4	0	0	0	0	0
12	4	0.05	0.2	0	0	0	0	0
13	3	0.05	0.1	0	0	0	0	0.1
14	2	0.06	0.01	0	0	0	0	0.01
15	2	0.03	0.1	0	0	0	0	0
16	1	0.01	0.1	0	0	0	0	0
17	1	0	0.01	0	0	0	0	0
18	0.4	0.02	0	0	0	0	0	0
19	0.2	0.01	0	0	0	0	0	0
20	0.1	0	0	0	0	0	0	0
≥20	0.6	0	0	0	0	0	0	0

### 5999 SNPs identified and characterized

**Fig. 4:**
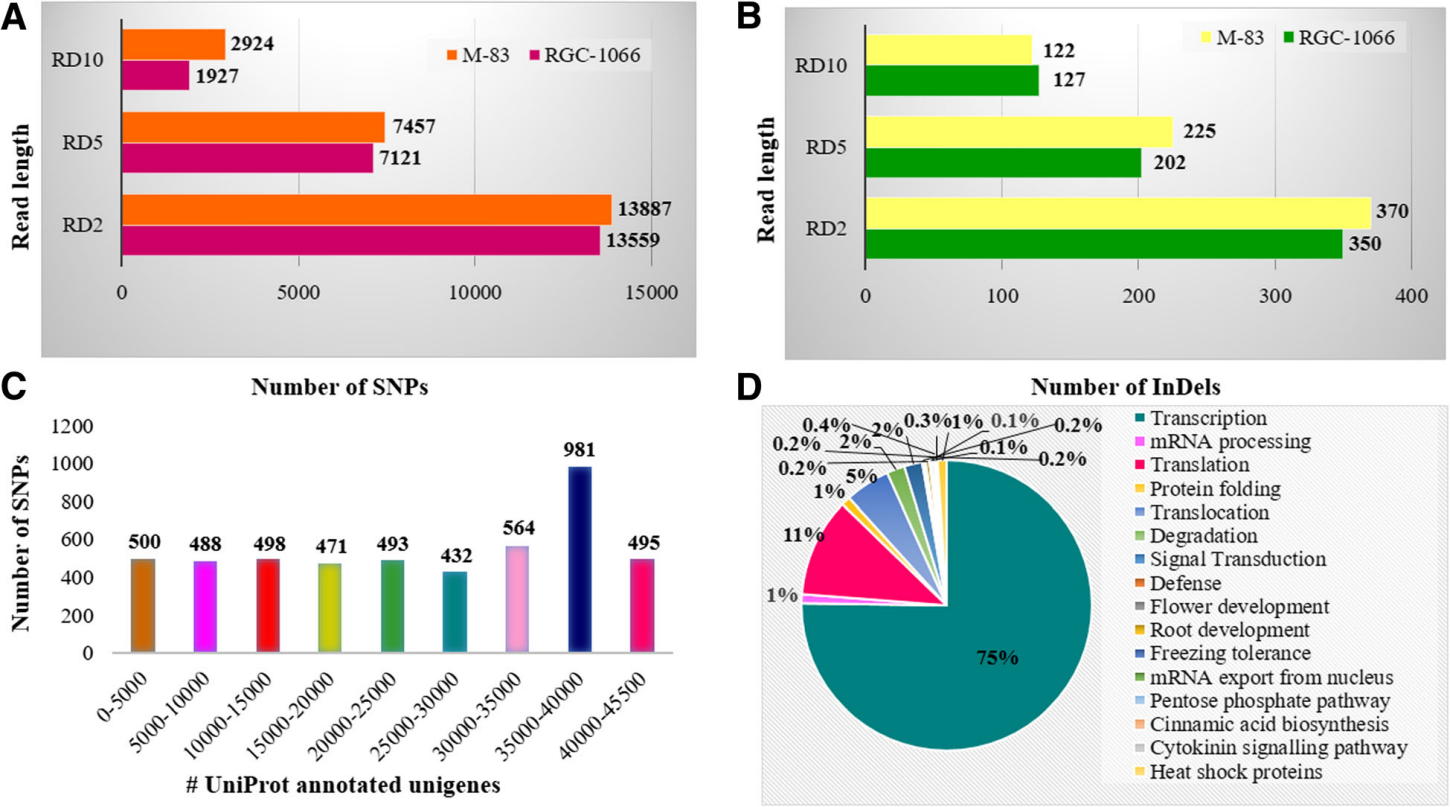
Number and frequency distributions of nucleotide-level variants in root transcriptomes of RGC-1066 and M-83 guar varieties **a**. SNP distribution at read depths (RD) 2, 5 and 10 **b**. InDels distribution at read depths (RD) 2, 5 and 10 **c**. SNPs distribution in the UniProt annotated unigenes **d**. Frequency distribution of SNPs in various gene functions of unigenes

A total of 27,446, 14,578 and 5999 single nucleotide polymorphisms (SNPs) were identified at read depths (RDs) of 2, 5 and 10, respectively, in the RGC-1066 and M-83 varieties of guar (Fig 4a, c). On an average, 1 SNP per 17.08 kb was found in the unigenes, at RD10. The SNPs obtained at higher RDs are considered to be high-confidence SNPs as compared to the ones obtained at lower RDs. Hence the SNPs obtained at the RD of 10 were used for further study. At this RD, the varieties RGC-1066 and M-83 were found to contain 2479 and 2870 SNPs, respectively (Additional file 17: Table S15). The SNP names, description/annotation of the SNP-containing unigenes, and alleles and positions of 4851 SNPs have been given in the Additional file 18: Table S16. These SNPs were found to be located in 2400 unigenes. The highest number of SNPs was found in the RNA dependent RNA polymerase (506) unigene. A total of 86 SNPs were found in 47 transcription factors which are involved in intracellular signaling, stress tolerance, regulation of cell cycle, cell growth, apoptosis and defense mechanisms. The LOC101508115 and Gag-Pol polyprotein unigenes, which are involved in nucleic acid binding and DNA integration, contained 58 and 49 SNPs, respectively.

The transition and transversion analysis of the SNPs has been presented in the Table 5. The total number of transition and transversion mutations were 3033 (62%) and 1803 (38%), respectively, with a Ts/Tv ratio of 1.72 (Table 5). Among the transition mutations, T/C and A/G showed high occurrences of 18.7 and 17.8%, respectively. The A/T and T/A mutations had high occurrences of 6.1 and 5.6%, respectively, among the transversion mutations. Five SNPs, namely, OT2, OT3, OT105, OT243 and OT4846, randomly selected for validation showed PCR amplification (Additional file 16: Figure S2B).

**Table 5:** Transition vs transversion mutation rate, type and ratio determined from the transcriptomes of RGC-1066 and M-83 varieties of guar

Transition type	Occurance/Number^a^	Percent occurance (%)
A/G	865	17.8
C/T	585	12.04
G/A	673	13.9
T/C	910	18.7
Transversion type	Occurance/Number^a^	Percent occurance (%)
A/C	206	4.2
A/T	297	6.1
C/G	202	4.1
G/T	214	4.4
C/A	232	4.8
T/A	275	5.6
G/C	140	2.9
T/G	237	4.9
Transition/Transversion ratio: 1.72		

^a^Out of 4907 SNPs

Sixteen SNPs were found to be located in 6 unigenes associated with root development (Table 6; Additional file 19: Table S17; Fig 4d). These unigenes encode for cellulose synthase A and D5 like proteins which are involved in mannan synthesis, root hair morphogenesis and root cell wall biosynthesis. Nine transition and 7 transversion mutations were found in these six unigenes.

**Table 6:** Characteristics of the SNPs located in genes involved in root development of guar

S. No.	SNP name	Gene name	SNP Allele	Position	Gene description	Gene function
1	OT3537	KK1_009388/ LOC100798193 GLYMA_06G069600	G/T	594	cellulose synthase A catalytic subunit 1 [UDP-forming]	Transportation of mannose, nucleotide-sugar transport
2	OT2789	-do-	C/A	898	-do-	β (1,4) Mannan synthesis
3	OT2790	-do-	C/A	899	-do-	-do-
4	OT2791	-do-	T/C	917	-do-	-do-
5	OT2792	-do-	A/G	959	-do-	-do-
6	OT2793	-do-	T/C	960	-do-	-do-
7	OT2794	-do-	G/T	1322	-do-	-do-
8	OT4846	LOC100797597 GLYMA_03G217500	T/A	344	cellulose synthase-like protein D5	-do-
9	OT1382	LR48_Vigan02g201500	T/A	901	cellulose synthase A catalytic subunit 2 [UDP-forming]-like	-do-
10	OT3553	LOC100789704 GLYMA_12G002500	A/C	1528	receptor-like protein kinase HSL1-like isoform X1	Regulation of root development
11	OT3554	-do-	A/G	3027	-do-	-do-
12	OT3555	-do-	A/G	3037	-do-	-do-
13	OT3861	LOC100780184 GLYMA_08G111400	A/G	2864	uncharacterized protein LOC100780184	Root cell wall biosynthesis
14	OT3862	-do-	A/G	5661	-do-	-do-
15	OT3863	-do-	A/G	7007	-do-	-do-
16	OT4656	LOC100795671 GLYMA_12G192000	T/C	2145	cellulose synthase-like protein B3-like	Root hair morphogenesis

A total of 27 SNPs were found in 14 unigenes involved in defense responses, hypersensitive responses, negative regulation of p53, heat and cold stress responses (Table 7; Fig 4d; Additional file 20: Table S18). These unigenes, encoding for NB-LRR, NB-ARC, transcription factors, heat shock proteins, ring finger and zinc finger domain containing proteins, were found to contain 13 transition and 14 transversion mutations.

**Table 7:** Characteristics of SNPs located in guar unigenes involved in biotic and abiotic stress responses

S. No.	SNP name	Gene name	SNP allele	SNP position	Gene description	Gene function
1	OT769	MTR_2g078070	A/G	3008	NBS-LRR type	Defense response
2	OT1113	KK1_009739	G/T	272	Disease resistance protein RGA2	Late blight (*Phytophthora infestans*) resistance
3	OT1114	-do-	A/G	428	-do-	-do-
4	OT1115	-do-	A/G	485	-do-	-do-
5	OT1116	-do-	A/C	1845	-do-	-do-
6	OT1161	MTR_3g035960	A/T	978	NBS-LRR type disease resistance protein	R (Resistance) genes, plant defense
7	OT1162	-do-	T/C	1196	-do-	-do-
8	OT1163	-do-	A/G	2357	-do-	-do-
9	OT1431	KK1_033834	C/T	493	Disease resistance RPP13-like protein 1	Plant hypersensitive response, disease resistance
10	OT1432	-do-	A/G	528	-do-	-do-
11	OT1433	-do-	T/G	548	-do-	-do-
12	OT1434	-do-	C/A	549	-do-	-do-
13	OT1435	-do-	T/G	550	-do-	-do-
14	OT1436	-do-	T/A	593	-do-	-do-
15	OT1437	-do-	G/A	1323	-do-	-do-
16	OT1881	MTR_3g466750	C/G	1389	NB-ARC domain disease resistance protein	Defense response
17	OT1916	MTR_3g020890	A/G	290	NB-LRR type disease resistance protein Rps1-k-2	*Phytophthora* resistance
18	OT2923	KK1_021617		588	S-norcoclaurine synthase	Pathogen related (PR) 10 protein, defense response
19	OT2924	-do-	C/G	648	-do-	-do-
20	OT3720	MTR_2g020040	G/A	876	RING finger and CHY zinc finger domain-containing protein	Negative regulation of p53
21	OT67	KK1_005680	C/T	228	KK1_005680	Response to freezing
22	OT458	TCM_044345	G/A	2522	dnaJ protein homolog	Response to heat stress
23	OT543	KK1_034657	T/A	718	Heat shock factor protein HSF24	Response to heat stress
24	OT3934	LOC100818430 GLYMA_13G105700	A/T	218	heat stress transcription factor A-2-like	Response to heat stress
25	OT3935	-do-	G/T	590	-do-	-do-
26	OT1691	KK1_001069	A/T	79	Heat stress transcription factor A-5	Response to heat stress
27	OT1738	LOC100820566 GLYMA_13G225700	C/T	146	Heat stress transcription factor A-4a-like	Response to heat stress

Fourteen SNPs were located in 7 unigenes involved in galactomannan synthesis (Table 8). Six SNPs were found in the KK1_009388 gene encoding cellulose synthase A catalytic subunit 1 involved in biosynthesis of mannan. These unigenes conatained 5 transitions and 9 transversions.

**Table 8:** Characteristics of the SNPs located in genes involved in galactomannan synthesis in guar

S. No.	SNP name	SNP allele	Type of SNP	Position	Gene name	Gene description	Function
1	OT697	T/A	TV	1856	LOC106775241	PREDICTED: callose synthase 11-like [*Vignaradiata* var. *radiata*]	Control delivery of UDP-Glc to the synthase
2	OT698	A/G	TT	4865	-do-	-do-	-do-
3	OT1715	C/G	TV	5925	glysoja_027079	Callose synthase 3 [*Glycine soja*]	-do-
4	OT4075	A/T	TV	2011	KK1_031323	Callose synthase 3 [*Cajanuscajan*]	-do-
5	OT4076	C/G	TV	3624	-do-	-do-	-do-
6	OT4342	T/C	TT	6780	LOC100787540 GLYMA_10G295100	PREDICTED: callose synthase 9-like isoform 1 [*Glycine max*]	-do-
7	OT1366	T/A	TV	901	LR48_Vigan02g201500	PREDICTED: cellulose synthase A catalytic subunit 2 [UDP-forming]-like [*Vignaangularis*]	-do-
8	OT2742	C/A	TV	898	KK1_009388	Cellulose synthase A catalytic subunit 1 [UDP-forming] [*Cajanuscajan*]	Mannan biosynthesis
9	OT2743	C/A	TV	899	-do-	-do-	-do-
10	OT2744	T/C	TT	917	-do-	-do-	-do-
11	OT2745	A/G	TT	959	-do-	-do-	-do-
12	OT2746	T/C	TT	960	-do-	-do-	-do-
13	OT2747	G/T	TV	1322	-do-	-do-	-do-
14	OT3490	G/T	TV	594	LOC100798193 GLYMA_06G069600	hypothetical protein GLYMA_06G069600 [Glycine max]	-do-

*Abbreviations: TT* Transition, *TV* Transversion, *UDP-Glc* Uridine diphosphate glucose

### Identification and characterization of 249 InDel markers

A total of 720, 427 and 249 InDels (insertions+deletions) were recorded in the RGC-1066 and M-83 varieties of guar at RDs 2, 5 and 10, respectively (Fig 4b). Among the high-confidence 249 InDels, 160 (64.26%) were insertions whereas 89 (35.74%) were deletion mutations (Additional file 21: Table S19). Each of the two guar varieties was found to contain more insertions as compared to deletions. The main characteristics of 249 high-confidence InDels have been given in Additional file 19: Table S17. On an average, one insertion per 282.5 kb was found in the annotated unigenes. The high-confidence 160 insertions were found to be located in 153 unigenes. The average size of an insertion was 1.49 bp with the range of 1–17 bp. The longest insertion of 17 bp was found to be present in LOC101504041 gene involved in N-acetyltransferase activity. On an average, one deletion was found per 507.9 kb in the annotated unigenes. These deletions were distributed in 87 unigenes. The average size of a deletion was 3.02 bp with variations of 1-18 bp. The longest deletion of 18 bp was located in the LOC100793810 GLYMA_07G170000 gene involved in tRNA methyltransferase activity and tRNA methylation.

### Differrential gene expression and metabolic network analyses

**Fig. 5:**
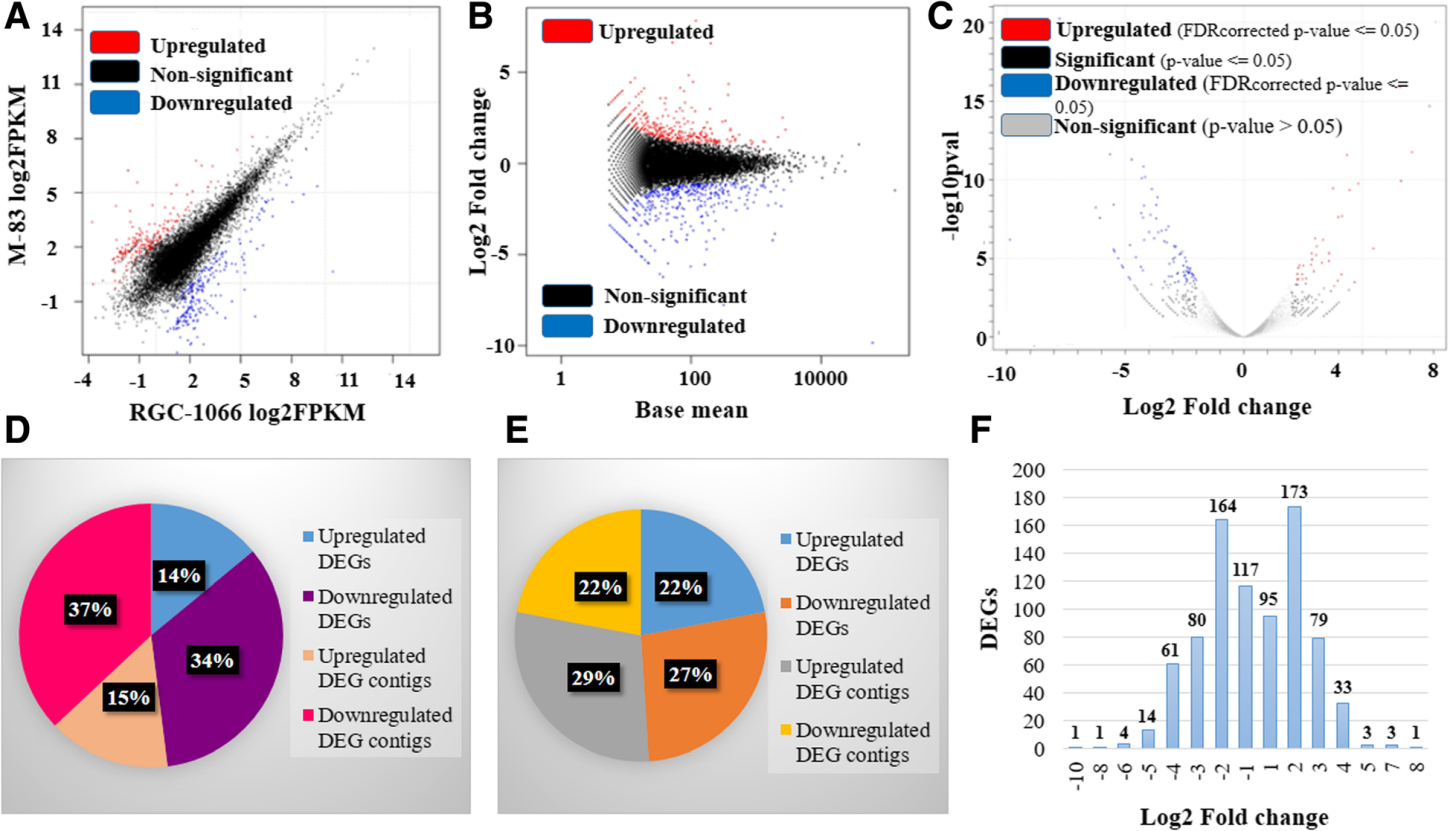
Distribution of differentially expressed genes in the roots of RGC-1066 and M-83 varieties of guar (**a**) FPKM plot (**b**) MA plot (log2Fold change vs Base mean) (**c**) Volcano plot (−log10pval vs log2Foldchange) (**d**) Pie padj plot (**e**) Pie pval plot (**f**) Bar plot of log2foldchange of upregulated, downregulated and non-significant unigenes

**Fig. 6:**
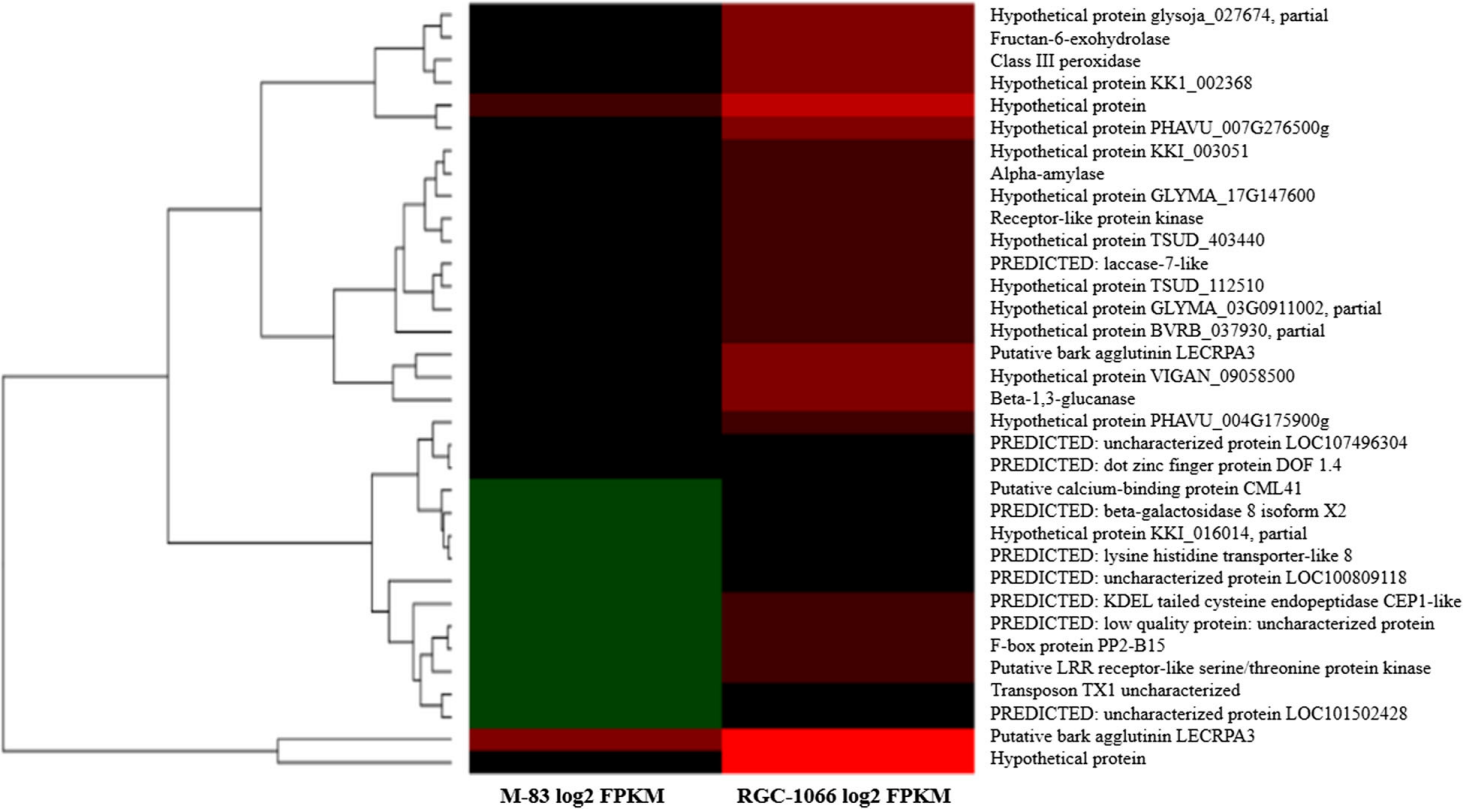
Heat-map representation of highly upregulated and downregulated differentially expressed genes in the roots of RGC-1066 and M-83 varieties of guar

Out of 404 unigenes (Benjamini-Hochberg adjusted *p*-value < 0.05) differentially expressed by more than two-fold in the two studied varieties of guar, 227 and 177 unigenes were found to be upregulated in the RGC-1066 and M-83 varieties, respectively (Figs 5, 6; Additional file 22: Table S20, Additional file 23: Table S21). The Fig 6 shows the top 20% of differentially expressed genes of RGC-1066 and M-83 varieties and the hierarchical distances of these genes. The detailed description of the differentially expressed genes has been presented in the Additional file 22: Table S20, Additional file 23: Table S21.

## Discussion

Genetic improvement of the industrially important guar crop has been so far accomplished following the traditional plant breeding approaches without using molecular markers. A molecular plant breeding programme requires a rich resource of molecular markers which is lacking for the guar crop. As the genome of guar has not been sequenced yet, obtaining such markers is not easy.

Among the various molecular markers, SSRs, SNPs and InDels are considered to be very valuable. Recently, development of SSR [13, 15–17] and SNP [16] markers has been reported in guar. However, these markers are less in number in comparison to those available for the model crops, and do not cover all the expressed regions of guar genome. Further, these markers have not been well characterized. We report here the development and characterization of SSR, SNP and InDel markers for the guar crop by sequencing of the root transcriptome.

By de novo assembly and clustering of high quality sequence reads obtained from RNA-Seq of roots of two guar varieties, RGC-1066 and M-83 having contrasting phenotypic characters, we generated 102,479 unigenes. From the RNA-Seq of leaf tissues of these two varieties, Tanwar et al. [16] reported the generation of 62,146 unigenes. Rawal et al. [17] obtained 127,706 unigenes from the RNA-Seq of three tissues, namely, leaves, shoots and flowers of guar variety RGC936. The comparisons of our sequence data with those of the above two reports indicates that the unigenes obtained by us have a good coverage of guar root transcriptome which is a prerequite for obtaining sufficient number of genic markers covering most of the genes. The CEGMA analysis showed that the unigenes obtained had 86.55% complete and 97.71% partial CEGs, indicating that our collection of unigenes was suitable for obtaining a sufficient number of molecular markers for a wide variety of guar genes expressing in roots [31–33]. Similar results have been obtained in the leaf transcriptomic analysis of guar [16]. Rawal et al. [17] have, however, reported more completedness in their transcriptomic library derived from the flower, leaf and shoot tissues of guar.

As the guar genome has not been sequenced, sequence similarity search of the 102,479 assembled unigenes was carried out by using BLASTX against six databases. A total of 54,249 unigenes had at least one significant match in blast hit results with E-value < 10^− 6^ showing that these unigenes are protein coding. The main reason for not getting hits for the remaining unigenes is that very low amounts of genomic data is available for guar in the known databases. These unigenes appear to be guar specific and may be useful for further research to obtain novel gene products, introduce new traits in other organisms and understand some biological mechanisms at the molecular level. The highest similarity (45.65%) of the assembled unigenes was found with *G. max* genes. Tanwar et al. [16] also reported 41.91% similarity of the guar unigenes with the genes of *G. max*. The leaf and root transcriptomic analyses of guar shows that *G. max* may be used as a suitable reference species for guar genetic analysis.

By screening of 102,479 unigenes assembled from the RNA-Seq data of two guar varieties, we obtained 25,040 high-confidence molecular markers which consist of 18,792 SSRs, 5999 SNPs and 249 InDels. The validation of random samples of these markers indicated that PCR amplification of most of these markers would be possible. The molecular markers identified by us have tagged most of the genes involved in root development, stress tolerance and other general metabolic activities. Each molecular marker has been characterized, particularly with respect to its precise position in the unigene containing it. For about 70% of the markers, we have been able to determine the characteristics of the genes containing these markers. We hope that the high-confidence molecular markers obtained by us, along with the others previously reported, will be helpful in studying molecular mechanisms of root development, stress tolerance and gum synthesis in guar. These markers are expected to contribute significantly to the breeding programmes based on marker-assisted selection, to develop improved varieties of guar.

Although we have enriched the guar molecular markers resource, this resource is still less than those available for the well-studied legume crops like soybean, *Lotus*, *Medicago*, pigeonpea and chickpea. Further research work, similar to ours, is needed to analyze the RNA-Seq data of other tissues of guar to obtain more molecular markers. The newly discovered molecular markers have to be characterized thoroughly. Genotyping-by-sequencing (GBS) would be better for a segregating population to obtain a high-density linkage map. The availability of the complete genomic sequence of guar will be highly helpful for the genetic improvement of this important crop.

## Conclusions

A total of 102,479 unigenes were assembled from the RNA-Seq data of two guar varieties, namely, RGC-1066 and M-83. The screening of these unigenes resulted in the identification of 25,040 high-confidence molecular markers which consisted of 18,792 SSRs, 5999 SNPs and 249 InDels. These molecular markers have been found to tag most of the genes involved in root development, stress tolerance and other general metabolic activities. All the molecular markers have been characterized, particularly with respect to their positions in the unigenes. We could determine the names, products and functions of the unigenes for 71% of the molecular markers.

## Methods

### Plant material and growth conditions

The seeds of RGC-1066 and M-83 varieties of guar were obtained from Rajasthan Agricultural Research Institute, Durgapura, Jaipur (India). The RGC-1066 is a commercial gum-producing variety having hairy leaf surface and purple colour flowers. The beans of the M-83 variety are used as a vegetable. This variety has glabrous leaves and white flowers. The plants were grown under field conditions at Indian Institute of Technology Roorkee. The root samples of each variety were collected on the 25th day of seed sowing and sent to SciGenome Labs Pvt. Ltd., Cochin (India) for RNA-Seq.

### Transcriptome sequencing

Three independent biological and technical replicates were used for cDNA library preparation and sequencing. The total RNA was extracted from the roots of each guar variety using Ambion^R^ Plant RNA isolation kit and cDNA library was prepared using TruSeq Stranded Total RNA Sample Prep Guide. The sequencing of cDNA library was carried out on Illumina HiSeq 2500 machine to obtain the raw reads. As per the sequencing strategy of the machine, the average read length was 100 bp.

### De novo assembly, clustering and functional annotation

The adaptor and repetitive sequences from raw reads were removed by Trimmomatic, NGS-pipe and Fast QC v0.11.5 softwares [34] to generate clean reads. The clean reads from three independent biological replicates of each variety were assembled into a fastafile by using the Trinity program.The reads in a fastafile are known as unigenes. The clustering and quality checking of unigenes were done using CD-HIT *v*4.6.6 [35] and CEGMA (Core Eukaryotic Gene Mapping Approach) [16]. Functional annotation of guar root transcriptome was done using BLASTX tool of BLAST2GO suite. Homologs of assembled unigenes were searched in NCBI, UniProt, Pathway, Nt, Pfam and Uniref90 databases with default parameters. The BLAST+ results were used to retrieve Gene Ontology (GO), and enzyme code (EC) terms using BLAST2GO suite. The functional and comparative analysis of othe assembled unigenes was done using TRAPID tool [36] to find closely related species. To understand the molecular datasets obtained at biological and functional level, the assembled unigenes were further annotated with KAAS (KEGG Automatic Annotation Server: http://www.genome..jp/kegg/kaas/) [37].

### Mining, validation and in silico analysis of SSRs

The mining of mono-, di-, tri-, tetra-, penta-, hexa-, and compound SSR markers was done using MISA tool, followed by validation and in silico analysis as given in [16]. Twenty SSR markers were randomly selected for validation. The primers were designed for these markers using Primer3 tool [38]. PCR with the above primers was carried out on the DNA samples extracted from the roots of RGC-1066 and M-83 guar varieties using DNeasy Plant Mini Kit (QIAGEN). The amplified PCR products were visualized on 3% agarose gel.

### Identification of single nucleotide polymorphisms (SNPs) and insertions and deletions (InDels)

The high quality clean transcriptomic sequence reads of two guar varieties RGC-1066 and M-83 were mapped on to the assembled unigenes using Bowtie2 version 2.3.2 software [39] (http://bowtie-bio.sourceforge.net/index.shtml) to obtain BAM files. These files were used to identify each read that mapped to only one position of the reference genome (de novo assembly). The FastQC version 0.11.5 tool (http://www.bioinformatics.babraham.ac.uk/projects/fastqc/) was used for base quality filtration. The identification of SNPs and InDels was done using SAMtools version 1.4 software [40], by setting default parameters at read depths 2, 5 and 10. The SNPs and InDels obtained were manually characterized.

### Validation of SNPs by AS-PCR (allele-specific PCR)

Allele specific PCR [41] was performed for the validation of SNPs. The DNA was extracted by CTAB method from the root of a 25 day old guar plant. Three primers, namely, forward, reverse and allele specific, were designed for amplification of each gene. The PCR amplification was performed using Applied Biosystem Veriti 96-well thermal cycler in a 20 μl reaction volume containing 150 ng of genomic DNA, 1X Taq buffer, 2 mM MgCl_2,_ 0.2 mM dNTP, 0.5 mM primers and 1 unit of Taq Polymerase. The gene sequences were amplified from genomic DNA of each guar variety at initial denaturation of 94 °C for 5 mins, 30 cycles of denaturation at 94 °C for 30s, annealing at 60 °C for 45 s, extension at 72 °C for 60s and final extension of 10 min at 72 °C. The amplified PCR products were visualized on 1.5% agarose gel and documented on gel documentation unit (Bio-Rad).

### Identification of differentially expressed genes

The differential gene expression and metabolic network analyses of unigenes of RGC-1066 and M-83 varieties of guar were performed using DeSeq2 program (http://www.bioconductor.org/packages/release/bioc/html/DESeq2.html) [42] and plant metabolic network database (http://www.plantcyc.org).

## Additional files

Additional file 1: **Table S1.** Number of transcripts and GC % of transcripts generated after de novo assembly of clean reads. (DOCX 11 kb)

Additional file 2: **Table S2.** Statistics of CEGMA results of guar root transcriptome assembly. (DOCX 12 kb)

Additional file 3: **Figure S1.** BLASTX distribution of unigenes obtained from root transcriptome of guar in different plant species. (TIF 410 kb)

Additional file 4: **Table S3.** Distribution classification of gene ontology (GO) terms in 45200 guar root unigenes. (XLSX 184 kb)

Additional file 5: **Table S4.** Pathway mapping of annotated unigenes of RGC-1066 and M-83 varieties of guar using KAAS-KEGG automatic annotation server. (DOCX 51 kb)

Additional file 6: **Table S5.** List of characteristics of 18972 SSR markers obtained from root transcriptome of guar. (XLS 2191 kb)

Additional file 7: **Table S6.** Characteristics of mononucleotide SSRs obtained in RGC-1066 and M-83 varieties of guar. (XLSX 391 kb)

Additional file 8: **Table S7.** Characteristics of dinucleotide SSRs obtained in RGC-1066 and M-83 varieties of guar. (XLSX 142 kb)

Additional file 9: **Table S8.** Characteristics of trinucleotide SSRs obtained in RGC-1066 and M-83 varieties of guar. (XLSX 177 kb)

Additional file 10: **Table S9.** Characteristics of tetranucleotide SSRs obtained in RGC-1066 and M-83 varieties of guar. (XLSX 22 kb)

Additional file 11: **Table S10.** Characteristics of pentanucleotide SSRs obtained in RGC-1066 and M-83 varieties of guar. (XLSX 11 kb)

Additional file 12: **Table S11.** Characteristics of hexanucleotide SSRs obtained in RGC-1066 and M-83 varieties of guar. (XLSX 12 kb)

Additional file 13: **Table S12.** Characteristics of compound SSRs obtained in RGC-1066 and M-83 varieties of guar. (XLSX 60 kb)

Additional file 14: **Table S13.** Characteristics of compound* SSRs obtained in RGC-1066 and M-83 varieties of guar. (XLSX 10 kb)

Additional file 15: **Table S14.** Frequency distribution of identified SSR repeats in RGC-1066 and M-83 guar varieties. (XLSX 13 kb)

Additional file 16: **Figure S2A.** PCR amplification results of SSR markers. Fig S2B. PCR amplification results of SNP markers. (TIF 1146 kb)

Additional file 17: **Table S15.** List of SNPs in RGC-1066 and M-83 guar varieties. (XLSX 2293 kb)

Additional file 18: **Table S16.** Characteristics of SNPs obtained at RD10 from root transcriptome of guar varieties RGC-1066 and M-83. (XLSX 356 kb)

Additional file 19: **Table S17.** List of the primers used to study the SNPs present in root development genes of guar. (DOCX 12 kb)

Additional file 20: **Table S18.** List of the primers used to study the SNPs located in guar unigenes involved in biotic and abiotic stress responses. (DOCX 13 kb)

Additional file 21: **Table S19.** Characteristics of InDels obtained at RD10 from root transcriptome of guar varieties RGC-1066 and M-83. (XLSX 37 kb)

Additional file 22: **Table S20.** Detailed description of differentially expressed genes of RGC-1066 variety of guar. (XLS 443 kb)

Additional file 23: **Table S21.** Detailed description of differentially expressed genes of M-83 variety of guar. (XLS 442 kb)

## Abbreviations

AS-PCR: Allele-specific PCR
CEGMA: Core eukaryotic gene mapping approach
CEGs: Conserved eukaryotic genes
EC: Enzyme code
GO: Gene ontology
InDel: Insertion-deletion
ISSR: Inter simple sequence repeat
NSG: Next generation sequencing
PCR: Polymerase chain reaction
RAPD: Random amplified polymorphic DNA
rDNA: Ribosomal DNA
RNA-Seq: Ribonucleic acid sequence
SCAR: Sequence characterized amplified region
SNP: Single nucleotide polymorphism
SSR: Simple sequence repeat
