# Supplementary material for: Identification and characterization of SSR, SNP and InDel molecular markers from RNA-Seq data of guar (Cyamopsis tetragonoloba, L. Taub.) roots
Source: BMC Genomics. 2018 Dec 20;19:951. doi: 10.1186/s12864-018-5205-9 (PMC6302463; doi:10.1186/s12864-018-5205-9)
Supplement: Supplementary file 1 — Table S1. Number of transcripts and GC % of transcripts generated after de novo assembly of clean reads. (DOCX 11 kb) [file 12864_2018_5205_MOESM1_ESM.docx]

Table S1. Number of transcripts and GC % of transcripts generated after *de novo* assembly of clean reads.

| **CD-HIT** | **Before CD-HIT** | **After CD-HIT** |
| --- | --- | --- |
| No. of assembled transcripts | 1,22,206 | 1,02,479 |
| Mean GC % of transcripts | 40.01% | 39.82% |
| Longest transcript length | 16,844 | 16,844 |
