# Supplementary material for: Identification and characterization of SSR, SNP and InDel molecular markers from RNA-Seq data of guar (Cyamopsis tetragonoloba, L. Taub.) roots
Source: BMC Genomics. 2018 Dec 20;19:951. doi: 10.1186/s12864-018-5205-9 (PMC6302463; doi:10.1186/s12864-018-5205-9)
Supplement: Supplementary file 2 — Table S2. Statistics of CEGMA results of guar root transcriptome assembly. (DOCX 12 kb) [file 12864_2018_5205_MOESM2_ESM.docx]

**Table S2. Statistics of CEGMA results of guar root transcriptome assembly.**

|  | **Prots** | **% Completeness** | **Total** | **Average** | **% Ortho** |
| --- | --- | --- | --- | --- | --- |
| **Complete** | 217 | 86.55 | 490 | 2.35 | 72.19 |
| Group 1 | 54 | 85.51 | 121 | 2.65 | 76.16 |
| Group 2 | 63 | 91.13 | 110 | 2.45 | 74.12 |
| Group 3 | 46 | 82.36 | 130 | 2.30 | 70.12 |
| Group 4 | 54 | 87.21 | 129 | 2.03 | 68.34 |
|  |  |  |  |  |  |
| **Partial** | 241 | 97.71 | 490 | 2.80 | 82.12 |
| Group 1 | 60 | 98.97 | 166 | 2.91 | 85.45 |
| Group 2 | 68 | 100.00 | 174 | 2.83 | 83.14 |
| Group 3 | 54 | 94.31 | 162 | 2.62 | 80.05 |
| Group 4 | 59 | 97.56 | 159 | 2.85 | 79.85 |

*Prots represents the number of 248 ultra-conserved CEGs present in genome, % Completeness represents percentage of 248 ultra-conserved CEGs present, Total represents total number of CEGs present including putative orthologs, Average represents the average number of orthologs per CEG and % Ortho represents percentage of detected CEGs that have more than 1 ortholog.*
