# Supplementary figures and images for: Identification and characterization of SSR, SNP and InDel molecular markers from RNA-Seq data of guar (Cyamopsis tetragonoloba, L. Taub.) roots
Source: BMC Genomics. 2018 Dec 20;19:951. doi: 10.1186/s12864-018-5205-9 (PMC6302463; doi:10.1186/s12864-018-5205-9)

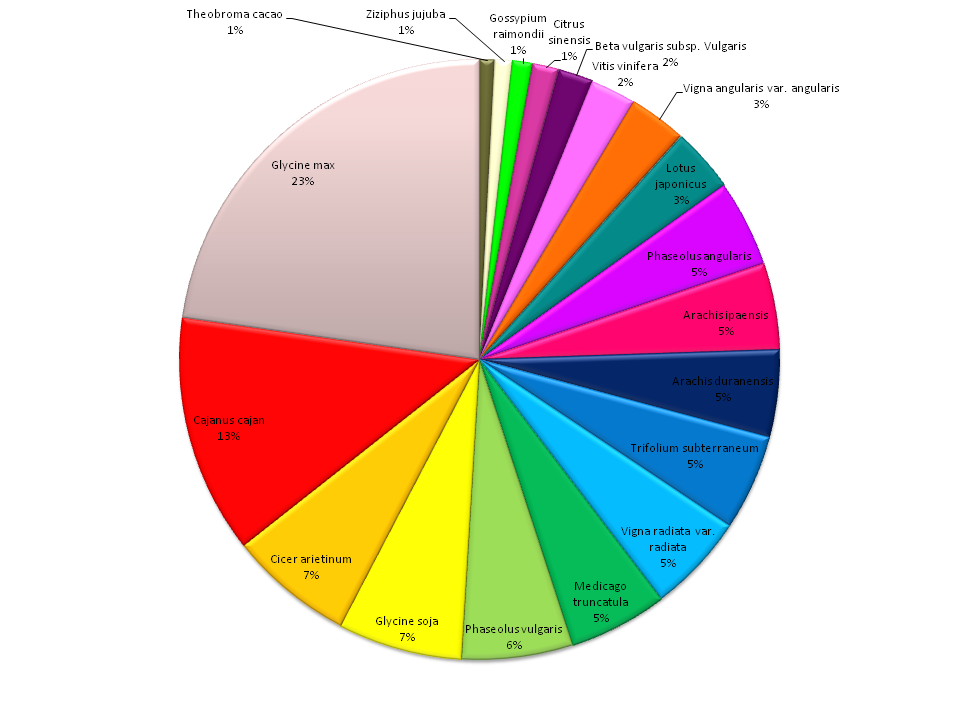

Supplement: Supplementary file 3 — Figure S1. BLASTX distribution of unigenes obtained from root transcriptome of guar in different plant species. (TIF 410 kb) [file 12864_2018_5205_MOESM3_ESM.tif]

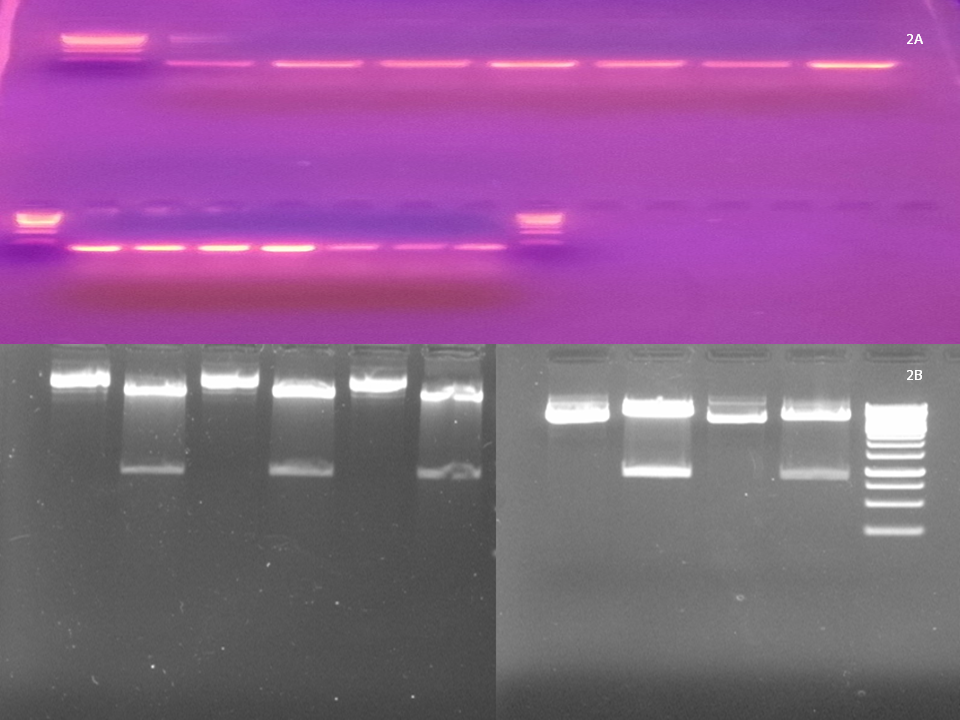

Supplement: Supplementary file 16 — Figure S2A. PCR amplification results of SSR markers. Fig S2B. PCR amplification results of SNP markers. (TIF 1146 kb) [file 12864_2018_5205_MOESM16_ESM.tif]
