# Supplementary material for: Identification and characterization of SSR, SNP and InDel molecular markers from RNA-Seq data of guar (Cyamopsis tetragonoloba, L. Taub.) roots
Source: BMC Genomics. 2018 Dec 20;19:951. doi: 10.1186/s12864-018-5205-9 (PMC6302463; doi:10.1186/s12864-018-5205-9)
Supplement: Supplementary file 5 — Table S4. Pathway mapping of annotated unigenes of RGC-1066 and M-83 varieties of guar using KAAS-KEGG automatic annotation server. (DOCX 51 kb) [file 12864_2018_5205_MOESM5_ESM.docx]

**Table S4. Pathway mapping of annotated unigenes of RGC-1066 and M-83 varieties of guar using KAAS-KEGG automatic annotation server.**

|  [ko01100 Metabolic pathways](https://www.genome.jp/kegg-bin/show_pathway?1531549175101874/ko01100.args) ([905](javascript:display('ko01100')))   [ko01110 Biosynthesis of secondary metabolites](https://www.genome.jp/kegg-bin/show_pathway?1531549175101874/ko01110.args) ([407](javascript:display('ko01110')))   [ko01120 Microbial metabolism in diverse environments](https://www.genome.jp/kegg-bin/show_pathway?1531549175101874/ko01120.args) ([157](javascript:display('ko01120')))   [ko01130 Biosynthesis of antibiotics](https://www.genome.jp/kegg-bin/show_pathway?1531549175101874/ko01130.args) ([211](javascript:display('ko01130')))   [ko01200 Carbon metabolism](https://www.genome.jp/kegg-bin/show_pathway?1531549175101874/ko01200.args) ([97](javascript:display('ko01200')))   [ko01210 2-Oxocarboxylic acid metabolism](https://www.genome.jp/kegg-bin/show_pathway?1531549175101874/ko01210.args) ([30](javascript:display('ko01210')))   [ko01212 Fatty acid metabolism](https://www.genome.jp/kegg-bin/show_pathway?1531549175101874/ko01212.args) ([28](javascript:display('ko01212')))   [ko01230 Biosynthesis of amino acids](https://www.genome.jp/kegg-bin/show_pathway?1531549175101874/ko01230.args) ([105](javascript:display('ko01230')))   [ko01220 Degradation of aromatic compounds](https://www.genome.jp/kegg-bin/show_pathway?1531549175101874/ko01220.args) ([4](javascript:display('ko01220')))   [ko00010 Glycolysis / Gluconeogenesis](https://www.genome.jp/kegg-bin/show_pathway?1531549175101874/ko00010.args) ([36](javascript:display('ko00010')))   [ko00020 Citrate cycle (TCA cycle)](https://www.genome.jp/kegg-bin/show_pathway?1531549175101874/ko00020.args) ([21](javascript:display('ko00020')))   [ko00030 Pentose phosphate pathway](https://www.genome.jp/kegg-bin/show_pathway?1531549175101874/ko00030.args) ([20](javascript:display('ko00030')))   [ko00040 Pentose and glucuronateinterconversions](https://www.genome.jp/kegg-bin/show_pathway?1531549175101874/ko00040.args) ([13](javascript:display('ko00040')))   [ko00051 Fructose and mannose metabolism](https://www.genome.jp/kegg-bin/show_pathway?1531549175101874/ko00051.args) ([25](javascript:display('ko00051')))   [ko00052 Galactose metabolism](https://www.genome.jp/kegg-bin/show_pathway?1531549175101874/ko00052.args) ([18](javascript:display('ko00052')))   [ko00053 Ascorbate and aldarate metabolism](https://www.genome.jp/kegg-bin/show_pathway?1531549175101874/ko00053.args) ([17](javascript:display('ko00053')))   [ko00500 Starch and sucrose metabolism](https://www.genome.jp/kegg-bin/show_pathway?1531549175101874/ko00500.args) ([38](javascript:display('ko00500')))   [ko00520 Amino sugar and nucleotide sugar metabolism](https://www.genome.jp/kegg-bin/show_pathway?1531549175101874/ko00520.args) ([44](javascript:display('ko00520')))   [ko00620 Pyruvate metabolism](https://www.genome.jp/kegg-bin/show_pathway?1531549175101874/ko00620.args) ([36](javascript:display('ko00620')))   [ko00630 Glyoxylate and dicarboxylate metabolism](https://www.genome.jp/kegg-bin/show_pathway?1531549175101874/ko00630.args) ([32](javascript:display('ko00630')))   [ko00640 Propanoate metabolism](https://www.genome.jp/kegg-bin/show_pathway?1531549175101874/ko00640.args) ([20](javascript:display('ko00640')))   [ko00650 Butanoate metabolism](https://www.genome.jp/kegg-bin/show_pathway?1531549175101874/ko00650.args) ([13](javascript:display('ko00650')))   [ko00660 C5-Branched dibasic acid metabolism](https://www.genome.jp/kegg-bin/show_pathway?1531549175101874/ko00660.args) ([5](javascript:display('ko00660')))   [ko00562 Inositol phosphate metabolism](https://www.genome.jp/kegg-bin/show_pathway?1531549175101874/ko00562.args) ([29](javascript:display('ko00562')))   [ko00190 Oxidative phosphorylation](https://www.genome.jp/kegg-bin/show_pathway?1531549175101874/ko00190.args) ([88](javascript:display('ko00190')))   [ko00195 Photosynthesis](https://www.genome.jp/kegg-bin/show_pathway?1531549175101874/ko00195.args) ([31](javascript:display('ko00195')))   [ko00196 Photosynthesis - antenna proteins](https://www.genome.jp/kegg-bin/show_pathway?1531549175101874/ko00196.args) ([7](javascript:display('ko00196')))   [ko00710 Carbon fixation in photosynthetic organisms](https://www.genome.jp/kegg-bin/show_pathway?1531549175101874/ko00710.args) ([24](javascript:display('ko00710')))   [ko00720 Carbon fixation pathways in prokaryotes](https://www.genome.jp/kegg-bin/show_pathway?1531549175101874/ko00720.args) ([16](javascript:display('ko00720')))   [ko00680 Methane metabolism](https://www.genome.jp/kegg-bin/show_pathway?1531549175101874/ko00680.args) ([22](javascript:display('ko00680')))   [ko00910 Nitrogen metabolism](https://www.genome.jp/kegg-bin/show_pathway?1531549175101874/ko00910.args) ([12](javascript:display('ko00910')))   [ko00920 Sulfur metabolism](https://www.genome.jp/kegg-bin/show_pathway?1531549175101874/ko00920.args) ([16](javascript:display('ko00920')))   [ko00061 Fatty acid biosynthesis](https://www.genome.jp/kegg-bin/show_pathway?1531549175101874/ko00061.args) ([16](javascript:display('ko00061')))   [ko00062 Fatty acid elongation](https://www.genome.jp/kegg-bin/show_pathway?1531549175101874/ko00062.args) ([8](javascript:display('ko00062')))   [ko00071 Fatty acid degradation](https://www.genome.jp/kegg-bin/show_pathway?1531549175101874/ko00071.args) ([14](javascript:display('ko00071')))   [ko00072 Synthesis and degradation of ketone bodies](https://www.genome.jp/kegg-bin/show_pathway?1531549175101874/ko00072.args) ([4](javascript:display('ko00072')))   [ko00073 Cutin, suberine and wax biosynthesis](https://www.genome.jp/kegg-bin/show_pathway?1531549175101874/ko00073.args) ([6](javascript:display('ko00073')))   [ko00100 Steroid biosynthesis](https://www.genome.jp/kegg-bin/show_pathway?1531549175101874/ko00100.args) ([18](javascript:display('ko00100')))   [ko00140 Steroid hormone biosynthesis](https://www.genome.jp/kegg-bin/show_pathway?1531549175101874/ko00140.args) ([5](javascript:display('ko00140')))   [ko00561 Glycerolipid metabolism](https://www.genome.jp/kegg-bin/show_pathway?1531549175101874/ko00561.args) ([29](javascript:display('ko00561')))   [ko00564 Glycerophospholipid metabolism](https://www.genome.jp/kegg-bin/show_pathway?1531549175101874/ko00564.args) ([39](javascript:display('ko00564')))   [ko00565 Ether lipid metabolism](https://www.genome.jp/kegg-bin/show_pathway?1531549175101874/ko00565.args) ([8](javascript:display('ko00565')))   [ko00600 Sphingolipid metabolism](https://www.genome.jp/kegg-bin/show_pathway?1531549175101874/ko00600.args) ([14](javascript:display('ko00600')))   [ko00590 Arachidonic acid metabolism](https://www.genome.jp/kegg-bin/show_pathway?1531549175101874/ko00590.args) ([9](javascript:display('ko00590')))   [ko00591 Linoleic acid metabolism](https://www.genome.jp/kegg-bin/show_pathway?1531549175101874/ko00591.args) ([4](javascript:display('ko00591')))   [ko00592 alpha-Linolenic acid metabolism](https://www.genome.jp/kegg-bin/show_pathway?1531549175101874/ko00592.args) ([14](javascript:display('ko00592')))   [ko01040 Biosynthesis of unsaturated fatty acids](https://www.genome.jp/kegg-bin/show_pathway?1531549175101874/ko01040.args) ([11](javascript:display('ko01040')))   [ko00230 Purine metabolism](https://www.genome.jp/kegg-bin/show_pathway?1531549175101874/ko00230.args) ([98](javascript:display('ko00230')))   [ko00240 Pyrimidine metabolism](https://www.genome.jp/kegg-bin/show_pathway?1531549175101874/ko00240.args) ([77](javascript:display('ko00240')))   [ko00250 Alanine, aspartate and glutamate metabolism](https://www.genome.jp/kegg-bin/show_pathway?1531549175101874/ko00250.args) ([29](javascript:display('ko00250')))   [ko00260 Glycine, serine and threonine metabolism](https://www.genome.jp/kegg-bin/show_pathway?1531549175101874/ko00260.args) ([34](javascript:display('ko00260')))   [ko00270 Cysteine and methionine metabolism](https://www.genome.jp/kegg-bin/show_pathway?1531549175101874/ko00270.args) ([42](javascript:display('ko00270')))   [ko00280 Valine, leucine and isoleucine degradation](https://www.genome.jp/kegg-bin/show_pathway?1531549175101874/ko00280.args) ([22](javascript:display('ko00280')))   [ko00290 Valine, leucine and isoleucine biosynthesis](https://www.genome.jp/kegg-bin/show_pathway?1531549175101874/ko00290.args) ([10](javascript:display('ko00290')))   [ko00300 Lysine biosynthesis](https://www.genome.jp/kegg-bin/show_pathway?1531549175101874/ko00300.args) ([9](javascript:display('ko00300')))   [ko00310 Lysine degradation](https://www.genome.jp/kegg-bin/show_pathway?1531549175101874/ko00310.args) ([16](javascript:display('ko00310')))   [ko00220 Arginine biosynthesis](https://www.genome.jp/kegg-bin/show_pathway?1531549175101874/ko00220.args) ([23](javascript:display('ko00220')))   [ko00330 Arginine and proline metabolism](https://www.genome.jp/kegg-bin/show_pathway?1531549175101874/ko00330.args) ([27](javascript:display('ko00330')))   [ko00340 Histidine metabolism](https://www.genome.jp/kegg-bin/show_pathway?1531549175101874/ko00340.args) ([13](javascript:display('ko00340')))   [ko00350 Tyrosine metabolism](https://www.genome.jp/kegg-bin/show_pathway?1531549175101874/ko00350.args) ([18](javascript:display('ko00350')))   [ko00360 Phenylalanine metabolism](https://www.genome.jp/kegg-bin/show_pathway?1531549175101874/ko00360.args) ([16](javascript:display('ko00360')))   [ko00380 Tryptophan metabolism](https://www.genome.jp/kegg-bin/show_pathway?1531549175101874/ko00380.args) ([16](javascript:display('ko00380')))   [ko00400 Phenylalanine, tyrosine and tryptophan biosynthesis](https://www.genome.jp/kegg-bin/show_pathway?1531549175101874/ko00400.args) ([25](javascript:display('ko00400')))   [ko00410 beta-Alanine metabolism](https://www.genome.jp/kegg-bin/show_pathway?1531549175101874/ko00410.args) ([17](javascript:display('ko00410')))   [ko00430 Taurine and hypotaurine metabolism](https://www.genome.jp/kegg-bin/show_pathway?1531549175101874/ko00430.args) ([3](javascript:display('ko00430')))   [ko00440 Phosphonate and phosphinate metabolism](https://www.genome.jp/kegg-bin/show_pathway?1531549175101874/ko00440.args) ([3](javascript:display('ko00440')))   [ko00450 Selenocompound metabolism](https://www.genome.jp/kegg-bin/show_pathway?1531549175101874/ko00450.args) ([10](javascript:display('ko00450')))   [ko00460 Cyanoamino acid metabolism](https://www.genome.jp/kegg-bin/show_pathway?1531549175101874/ko00460.args) ([10](javascript:display('ko00460')))   [ko00471 D-Glutamine and D-glutamate metabolism](https://www.genome.jp/kegg-bin/show_pathway?1531549175101874/ko00471.args) ([2](javascript:display('ko00471')))   [ko00473 D-Alanine metabolism](https://www.genome.jp/kegg-bin/show_pathway?1531549175101874/ko00473.args) ([1](javascript:display('ko00473')))   [ko00480 Glutathione metabolism](https://www.genome.jp/kegg-bin/show_pathway?1531549175101874/ko00480.args) ([19](javascript:display('ko00480')))   [ko00510 N-Glycan biosynthesis](https://www.genome.jp/kegg-bin/show_pathway?1531549175101874/ko00510.args) ([31](javascript:display('ko00510')))   [ko00513 Various types of N-glycan biosynthesis](https://www.genome.jp/kegg-bin/show_pathway?1531549175101874/ko00513.args) ([23](javascript:display('ko00513')))   [ko00515 Mannose type O-glycan biosynthesis](https://www.genome.jp/kegg-bin/show_pathway?1531549175101874/ko00515.args) ([2](javascript:display('ko00515')))   [ko00514 Other types of O-glycan biosynthesis](https://www.genome.jp/kegg-bin/show_pathway?1531549175101874/ko00514.args) ([7](javascript:display('ko00514')))   [ko00532 Glycosaminoglycan biosynthesis - chondroitin sulfate / dermatansulfate](https://www.genome.jp/kegg-bin/show_pathway?1531549175101874/ko00532.args) ([2](javascript:display('ko00532')))   [ko00534 Glycosaminoglycan biosynthesis - heparansulfate / heparin](https://www.genome.jp/kegg-bin/show_pathway?1531549175101874/ko00534.args) ([3](javascript:display('ko00534')))   [ko00531 Glycosaminoglycan degradation](https://www.genome.jp/kegg-bin/show_pathway?1531549175101874/ko00531.args) ([5](javascript:display('ko00531')))   [ko00563 Glycosylphosphatidylinositol (GPI)-anchor biosynthesis](https://www.genome.jp/kegg-bin/show_pathway?1531549175101874/ko00563.args) ([21](javascript:display('ko00563')))   [ko00601 Glycosphingolipid biosynthesis - lacto and neolacto series](https://www.genome.jp/kegg-bin/show_pathway?1531549175101874/ko00601.args) ([2](javascript:display('ko00601')))   [ko00603 Glycosphingolipid biosynthesis - globo and isoglobo series](https://www.genome.jp/kegg-bin/show_pathway?1531549175101874/ko00603.args) ([3](javascript:display('ko00603')))   [ko00604 Glycosphingolipid biosynthesis - ganglio series](https://www.genome.jp/kegg-bin/show_pathway?1531549175101874/ko00604.args) ([2](javascript:display('ko00604')))   [ko00540 Lipopolysaccharide biosynthesis](https://www.genome.jp/kegg-bin/show_pathway?1531549175101874/ko00540.args) ([8](javascript:display('ko00540')))   [ko00550 Peptidoglycan biosynthesis](https://www.genome.jp/kegg-bin/show_pathway?1531549175101874/ko00550.args) ([2](javascript:display('ko00550')))   [ko00511 Other glycan degradation](https://www.genome.jp/kegg-bin/show_pathway?1531549175101874/ko00511.args) ([10](javascript:display('ko00511')))   [ko00730 Thiamine metabolism](https://www.genome.jp/kegg-bin/show_pathway?1531549175101874/ko00730.args) ([13](javascript:display('ko00730')))   [ko00740 Riboflavin metabolism](https://www.genome.jp/kegg-bin/show_pathway?1531549175101874/ko00740.args) ([13](javascript:display('ko00740')))   [ko00750 Vitamin B6 metabolism](https://www.genome.jp/kegg-bin/show_pathway?1531549175101874/ko00750.args) ([9](javascript:display('ko00750')))   [ko00760 Nicotinate and nicotinamide metabolism](https://www.genome.jp/kegg-bin/show_pathway?1531549175101874/ko00760.args) ([13](javascript:display('ko00760')))   [ko00770 Pantothenate and CoA biosynthesis](https://www.genome.jp/kegg-bin/show_pathway?1531549175101874/ko00770.args) ([18](javascript:display('ko00770')))   [ko00780 Biotin metabolism](https://www.genome.jp/kegg-bin/show_pathway?1531549175101874/ko00780.args) ([8](javascript:display('ko00780')))   [ko00785 Lipoic acid metabolism](https://www.genome.jp/kegg-bin/show_pathway?1531549175101874/ko00785.args) ([2](javascript:display('ko00785')))   [ko00790 Folate biosynthesis](https://www.genome.jp/kegg-bin/show_pathway?1531549175101874/ko00790.args) ([24](javascript:display('ko00790')))   [ko00670 One carbon pool by folate](https://www.genome.jp/kegg-bin/show_pathway?1531549175101874/ko00670.args) ([11](javascript:display('ko00670')))   [ko00830 Retinol metabolism](https://www.genome.jp/kegg-bin/show_pathway?1531549175101874/ko00830.args) ([6](javascript:display('ko00830')))   [ko00860 Porphyrin and chlorophyll metabolism](https://www.genome.jp/kegg-bin/show_pathway?1531549175101874/ko00860.args) ([35](javascript:display('ko00860')))   [ko00130 Ubiquinone and other terpenoid-quinone biosynthesis](https://www.genome.jp/kegg-bin/show_pathway?1531549175101874/ko00130.args) ([23](javascript:display('ko00130')))   [ko00900 Terpenoid backbone biosynthesis](https://www.genome.jp/kegg-bin/show_pathway?1531549175101874/ko00900.args) ([29](javascript:display('ko00900')))   [ko00902 Monoterpenoid biosynthesis](https://www.genome.jp/kegg-bin/show_pathway?1531549175101874/ko00902.args) ([3](javascript:display('ko00902')))   [ko00909 Sesquiterpenoid and triterpenoid biosynthesis](https://www.genome.jp/kegg-bin/show_pathway?1531549175101874/ko00909.args) ([4](javascript:display('ko00909')))   [ko00904 Diterpenoid biosynthesis](https://www.genome.jp/kegg-bin/show_pathway?1531549175101874/ko00904.args) ([6](javascript:display('ko00904')))   [ko00906 Carotenoid biosynthesis](https://www.genome.jp/kegg-bin/show_pathway?1531549175101874/ko00906.args) ([19](javascript:display('ko00906')))   [ko00905 Brassinosteroid biosynthesis](https://www.genome.jp/kegg-bin/show_pathway?1531549175101874/ko00905.args) ([5](javascript:display('ko00905')))   [ko00981 Insect hormone biosynthesis](https://www.genome.jp/kegg-bin/show_pathway?1531549175101874/ko00981.args) ([1](javascript:display('ko00981')))   [ko00908 Zeatin biosynthesis](https://www.genome.jp/kegg-bin/show_pathway?1531549175101874/ko00908.args) ([4](javascript:display('ko00908')))   [ko00903 Limonene and pinene degradation](https://www.genome.jp/kegg-bin/show_pathway?1531549175101874/ko00903.args) ([1](javascript:display('ko00903')))   [ko00281 Geraniol degradation](https://www.genome.jp/kegg-bin/show_pathway?1531549175101874/ko00281.args) ([1](javascript:display('ko00281')))   [ko01051 Biosynthesis of ansamycins](https://www.genome.jp/kegg-bin/show_pathway?1531549175101874/ko01051.args) ([1](javascript:display('ko01051')))   [ko00523 Polyketide sugar unit biosynthesis](https://www.genome.jp/kegg-bin/show_pathway?1531549175101874/ko00523.args) ([2](javascript:display('ko00523')))   [ko00940 Phenylpropanoid biosynthesis](https://www.genome.jp/kegg-bin/show_pathway?1531549175101874/ko00940.args) ([18](javascript:display('ko00940')))   [ko00945 Stilbenoid, diarylheptanoid and gingerol biosynthesis](https://www.genome.jp/kegg-bin/show_pathway?1531549175101874/ko00945.args) ([4](javascript:display('ko00945')))   [ko00941 Flavonoid biosynthesis](https://www.genome.jp/kegg-bin/show_pathway?1531549175101874/ko00941.args) ([9](javascript:display('ko00941')))   [ko00944 Flavone and flavonol biosynthesis](https://www.genome.jp/kegg-bin/show_pathway?1531549175101874/ko00944.args) ([2](javascript:display('ko00944')))   [ko00942 Anthocyanin biosynthesis](https://www.genome.jp/kegg-bin/show_pathway?1531549175101874/ko00942.args) ([1](javascript:display('ko00942')))   [ko00901 Indole alkaloid biosynthesis](https://www.genome.jp/kegg-bin/show_pathway?1531549175101874/ko00901.args) ([3](javascript:display('ko00901')))   [ko00950 Isoquinoline alkaloid biosynthesis](https://www.genome.jp/kegg-bin/show_pathway?1531549175101874/ko00950.args) ([7](javascript:display('ko00950')))   [ko00960 Tropane, piperidine and pyridine alkaloid biosynthesis](https://www.genome.jp/kegg-bin/show_pathway?1531549175101874/ko00960.args) ([8](javascript:display('ko00960')))   [ko00232 Caffeine metabolism](https://www.genome.jp/kegg-bin/show_pathway?1531549175101874/ko00232.args) ([2](javascript:display('ko00232')))   [ko00965 Betalain biosynthesis](https://www.genome.jp/kegg-bin/show_pathway?1531549175101874/ko00965.args) ([2](javascript:display('ko00965')))   [ko00966 Glucosinolate biosynthesis](https://www.genome.jp/kegg-bin/show_pathway?1531549175101874/ko00966.args) ([3](javascript:display('ko00966')))   [ko00311 Penicillin and cephalosporin biosynthesis](https://www.genome.jp/kegg-bin/show_pathway?1531549175101874/ko00311.args) ([1](javascript:display('ko00311')))   [ko00332 Carbapenem biosynthesis](https://www.genome.jp/kegg-bin/show_pathway?1531549175101874/ko00332.args) ([2](javascript:display('ko00332')))   [ko00261 Monobactam biosynthesis](https://www.genome.jp/kegg-bin/show_pathway?1531549175101874/ko00261.args) ([6](javascript:display('ko00261')))   [ko00521 Streptomycin biosynthesis](https://www.genome.jp/kegg-bin/show_pathway?1531549175101874/ko00521.args) ([5](javascript:display('ko00521')))   [ko00524 Neomycin, kanamycin and gentamicin biosynthesis](https://www.genome.jp/kegg-bin/show_pathway?1531549175101874/ko00524.args) ([1](javascript:display('ko00524')))   [ko00525 Acarbose and validamycin biosynthesis](https://www.genome.jp/kegg-bin/show_pathway?1531549175101874/ko00525.args) ([1](javascript:display('ko00525')))   [ko00401 Novobiocin biosynthesis](https://www.genome.jp/kegg-bin/show_pathway?1531549175101874/ko00401.args) ([2](javascript:display('ko00401')))   [ko00405 Phenazine biosynthesis](https://www.genome.jp/kegg-bin/show_pathway?1531549175101874/ko00405.args) ([2](javascript:display('ko00405')))   [ko00333 Prodigiosin biosynthesis](https://www.genome.jp/kegg-bin/show_pathway?1531549175101874/ko00333.args) ([3](javascript:display('ko00333')))   [ko00254 Aflatoxin biosynthesis](https://www.genome.jp/kegg-bin/show_pathway?1531549175101874/ko00254.args) ([1](javascript:display('ko00254')))   [ko00362 Benzoate degradation](https://www.genome.jp/kegg-bin/show_pathway?1531549175101874/ko00362.args) ([2](javascript:display('ko00362')))   [ko00627 Aminobenzoate degradation](https://www.genome.jp/kegg-bin/show_pathway?1531549175101874/ko00627.args) ([2](javascript:display('ko00627')))   [ko00364 Fluorobenzoate degradation](https://www.genome.jp/kegg-bin/show_pathway?1531549175101874/ko00364.args) ([1](javascript:display('ko00364')))   [ko00625 Chloroalkane and chloroalkene degradation](https://www.genome.jp/kegg-bin/show_pathway?1531549175101874/ko00625.args) ([5](javascript:display('ko00625')))   [ko00361 Chlorocyclohexane and chlorobenzene degradation](https://www.genome.jp/kegg-bin/show_pathway?1531549175101874/ko00361.args) ([1](javascript:display('ko00361')))   [ko00623 Toluene degradation](https://www.genome.jp/kegg-bin/show_pathway?1531549175101874/ko00623.args) ([1](javascript:display('ko00623')))   [ko00643 Styrene degradation](https://www.genome.jp/kegg-bin/show_pathway?1531549175101874/ko00643.args) ([4](javascript:display('ko00643')))   [ko00791 Atrazine degradation](https://www.genome.jp/kegg-bin/show_pathway?1531549175101874/ko00791.args) ([2](javascript:display('ko00791')))   [ko00930 Caprolactam degradation](https://www.genome.jp/kegg-bin/show_pathway?1531549175101874/ko00930.args) ([1](javascript:display('ko00930')))   [ko00626 Naphthalene degradation](https://www.genome.jp/kegg-bin/show_pathway?1531549175101874/ko00626.args) ([2](javascript:display('ko00626')))   [ko00980 Metabolism of xenobiotics by cytochrome P450](https://www.genome.jp/kegg-bin/show_pathway?1531549175101874/ko00980.args) ([7](javascript:display('ko00980')))   [ko00982 Drug metabolism - cytochrome P450](https://www.genome.jp/kegg-bin/show_pathway?1531549175101874/ko00982.args) ([5](javascript:display('ko00982')))   [ko00983 Drug metabolism - other enzymes](https://www.genome.jp/kegg-bin/show_pathway?1531549175101874/ko00983.args) ([19](javascript:display('ko00983')))   [ko03020 RNA polymerase](https://www.genome.jp/kegg-bin/show_pathway?1531549175101874/ko03020.args) ([30](javascript:display('ko03020')))   [ko03022 Basal transcription factors](https://www.genome.jp/kegg-bin/show_pathway?1531549175101874/ko03022.args) ([30](javascript:display('ko03022')))   [ko03040 Spliceosome](https://www.genome.jp/kegg-bin/show_pathway?1531549175101874/ko03040.args) ([104](javascript:display('ko03040')))   [ko03010 Ribosome](https://www.genome.jp/kegg-bin/show_pathway?1531549175101874/ko03010.args) ([130](javascript:display('ko03010')))   [ko00970 Aminoacyl-tRNA biosynthesis](https://www.genome.jp/kegg-bin/show_pathway?1531549175101874/ko00970.args) ([27](javascript:display('ko00970')))   [ko03013 RNA transport](https://www.genome.jp/kegg-bin/show_pathway?1531549175101874/ko03013.args) ([96](javascript:display('ko03013')))   [ko03015 mRNA surveillance pathway](https://www.genome.jp/kegg-bin/show_pathway?1531549175101874/ko03015.args) ([52](javascript:display('ko03015')))   [ko03008 Ribosome biogenesis in eukaryotes](https://www.genome.jp/kegg-bin/show_pathway?1531549175101874/ko03008.args) ([58](javascript:display('ko03008')))   [ko03060 Protein export](https://www.genome.jp/kegg-bin/show_pathway?1531549175101874/ko03060.args) ([27](javascript:display('ko03060')))   [ko04141 Protein processing in endoplasmic reticulum](https://www.genome.jp/kegg-bin/show_pathway?1531549175101874/ko04141.args) ([77](javascript:display('ko04141')))   [ko04130 SNARE interactions in vesicular transport](https://www.genome.jp/kegg-bin/show_pathway?1531549175101874/ko04130.args) ([18](javascript:display('ko04130')))   [ko04120 Ubiquitin mediated proteolysis](https://www.genome.jp/kegg-bin/show_pathway?1531549175101874/ko04120.args) ([61](javascript:display('ko04120')))   [ko04122 Sulfur relay system](https://www.genome.jp/kegg-bin/show_pathway?1531549175101874/ko04122.args) ([10](javascript:display('ko04122')))   [ko03050 Proteasome](https://www.genome.jp/kegg-bin/show_pathway?1531549175101874/ko03050.args) ([35](javascript:display('ko03050')))   [ko03018 RNA degradation](https://www.genome.jp/kegg-bin/show_pathway?1531549175101874/ko03018.args) ([53](javascript:display('ko03018')))   [ko03030 DNA replication](https://www.genome.jp/kegg-bin/show_pathway?1531549175101874/ko03030.args) ([35](javascript:display('ko03030')))   [ko03410 Base excision repair](https://www.genome.jp/kegg-bin/show_pathway?1531549175101874/ko03410.args) ([26](javascript:display('ko03410')))   [ko03420 Nucleotide excision repair](https://www.genome.jp/kegg-bin/show_pathway?1531549175101874/ko03420.args) ([38](javascript:display('ko03420')))   [ko03430 Mismatch repair](https://www.genome.jp/kegg-bin/show_pathway?1531549175101874/ko03430.args) ([22](javascript:display('ko03430')))   [ko03440 Homologous recombination](https://www.genome.jp/kegg-bin/show_pathway?1531549175101874/ko03440.args) ([33](javascript:display('ko03440')))   [ko03450 Non-homologous end-joining](https://www.genome.jp/kegg-bin/show_pathway?1531549175101874/ko03450.args) ([8](javascript:display('ko03450')))   [ko03460 Fanconianemia pathway](https://www.genome.jp/kegg-bin/show_pathway?1531549175101874/ko03460.args) ([28](javascript:display('ko03460')))   [ko02010 ABC transporters](https://www.genome.jp/kegg-bin/show_pathway?1531549175101874/ko02010.args) ([14](javascript:display('ko02010')))   [ko03070 Bacterial secretion system](https://www.genome.jp/kegg-bin/show_pathway?1531549175101874/ko03070.args) ([7](javascript:display('ko03070')))   [ko02020 Two-component system](https://www.genome.jp/kegg-bin/show_pathway?1531549175101874/ko02020.args) ([23](javascript:display('ko02020')))   [ko04014 Rassignaling pathway](https://www.genome.jp/kegg-bin/show_pathway?1531549175101874/ko04014.args) ([12](javascript:display('ko04014')))   [ko04015 Rap1 signaling pathway](https://www.genome.jp/kegg-bin/show_pathway?1531549175101874/ko04015.args) ([8](javascript:display('ko04015')))   [ko04010 MAPK signaling pathway](https://www.genome.jp/kegg-bin/show_pathway?1531549175101874/ko04010.args) ([16](javascript:display('ko04010')))   [ko04013 MAPK signaling pathway - fly](https://www.genome.jp/kegg-bin/show_pathway?1531549175101874/ko04013.args) ([15](javascript:display('ko04013')))   [ko04016 MAPK signaling pathway - plant](https://www.genome.jp/kegg-bin/show_pathway?1531549175101874/ko04016.args) ([41](javascript:display('ko04016')))   [ko04011 MAPK signaling pathway - yeast](https://www.genome.jp/kegg-bin/show_pathway?1531549175101874/ko04011.args) ([17](javascript:display('ko04011')))   [ko04012 ErbBsignaling pathway](https://www.genome.jp/kegg-bin/show_pathway?1531549175101874/ko04012.args) ([8](javascript:display('ko04012')))   [ko04310 Wntsignaling pathway](https://www.genome.jp/kegg-bin/show_pathway?1531549175101874/ko04310.args) ([20](javascript:display('ko04310')))   [ko04330 Notch signaling pathway](https://www.genome.jp/kegg-bin/show_pathway?1531549175101874/ko04330.args) ([8](javascript:display('ko04330')))   [ko04340 Hedgehog signaling pathway](https://www.genome.jp/kegg-bin/show_pathway?1531549175101874/ko04340.args) ([5](javascript:display('ko04340')))   [ko04341 Hedgehog signaling pathway - fly](https://www.genome.jp/kegg-bin/show_pathway?1531549175101874/ko04341.args) ([9](javascript:display('ko04341')))   [ko04350 TGF-beta signaling pathway](https://www.genome.jp/kegg-bin/show_pathway?1531549175101874/ko04350.args) ([9](javascript:display('ko04350')))   [ko04390 Hippo signaling pathway](https://www.genome.jp/kegg-bin/show_pathway?1531549175101874/ko04390.args) ([8](javascript:display('ko04390')))   [ko04391 Hippo signaling pathway - fly](https://www.genome.jp/kegg-bin/show_pathway?1531549175101874/ko04391.args) ([8](javascript:display('ko04391')))   [ko04392 Hippo signaling pathway - multiple species](https://www.genome.jp/kegg-bin/show_pathway?1531549175101874/ko04392.args) ([3](javascript:display('ko04392')))   [ko04370 VEGF signaling pathway](https://www.genome.jp/kegg-bin/show_pathway?1531549175101874/ko04370.args) ([6](javascript:display('ko04370')))   [ko04371 Apelinsignaling pathway](https://www.genome.jp/kegg-bin/show_pathway?1531549175101874/ko04371.args) ([20](javascript:display('ko04371')))   [ko04630 JAK-STAT signaling pathway](https://www.genome.jp/kegg-bin/show_pathway?1531549175101874/ko04630.args) ([3](javascript:display('ko04630')))   [ko04064 NF-kappa B signaling pathway](https://www.genome.jp/kegg-bin/show_pathway?1531549175101874/ko04064.args) ([8](javascript:display('ko04064')))   [ko04668 TNF signaling pathway](https://www.genome.jp/kegg-bin/show_pathway?1531549175101874/ko04668.args) ([5](javascript:display('ko04668')))   [ko04066 HIF-1 signaling pathway](https://www.genome.jp/kegg-bin/show_pathway?1531549175101874/ko04066.args) ([17](javascript:display('ko04066')))   [ko04068 FoxOsignaling pathway](https://www.genome.jp/kegg-bin/show_pathway?1531549175101874/ko04068.args) ([20](javascript:display('ko04068')))   [ko04020 Calcium signaling pathway](https://www.genome.jp/kegg-bin/show_pathway?1531549175101874/ko04020.args) ([11](javascript:display('ko04020')))   [ko04070 Phosphatidylinositol signaling system](https://www.genome.jp/kegg-bin/show_pathway?1531549175101874/ko04070.args) ([24](javascript:display('ko04070')))   [ko04072 Phospholipase D signaling pathway](https://www.genome.jp/kegg-bin/show_pathway?1531549175101874/ko04072.args) ([14](javascript:display('ko04072')))   [ko04071 Sphingolipidsignaling pathway](https://www.genome.jp/kegg-bin/show_pathway?1531549175101874/ko04071.args) ([20](javascript:display('ko04071')))   [ko04024 cAMPsignaling pathway](https://www.genome.jp/kegg-bin/show_pathway?1531549175101874/ko04024.args) ([14](javascript:display('ko04024')))   [ko04022 cGMP-PKG signaling pathway](https://www.genome.jp/kegg-bin/show_pathway?1531549175101874/ko04022.args) ([12](javascript:display('ko04022')))   [ko04151 PI3K-Akt signaling pathway](https://www.genome.jp/kegg-bin/show_pathway?1531549175101874/ko04151.args) ([25](javascript:display('ko04151')))   [ko04152 AMPK signaling pathway](https://www.genome.jp/kegg-bin/show_pathway?1531549175101874/ko04152.args) ([27](javascript:display('ko04152')))   [ko04150 mTORsignaling pathway](https://www.genome.jp/kegg-bin/show_pathway?1531549175101874/ko04150.args) ([29](javascript:display('ko04150')))   [ko04075 Plant hormone signal transduction](https://www.genome.jp/kegg-bin/show_pathway?1531549175101874/ko04075.args) ([40](javascript:display('ko04075')))   [ko04080 Neuroactive ligand-receptor interaction](https://www.genome.jp/kegg-bin/show_pathway?1531549175101874/ko04080.args) ([2](javascript:display('ko04080')))   [ko04512 ECM-receptor interaction](https://www.genome.jp/kegg-bin/show_pathway?1531549175101874/ko04512.args) ([1](javascript:display('ko04512')))   [ko04144 Endocytosis](https://www.genome.jp/kegg-bin/show_pathway?1531549175101874/ko04144.args) ([57](javascript:display('ko04144')))   [ko04145 Phagosome](https://www.genome.jp/kegg-bin/show_pathway?1531549175101874/ko04145.args) ([28](javascript:display('ko04145')))   [ko04142 Lysosome](https://www.genome.jp/kegg-bin/show_pathway?1531549175101874/ko04142.args) ([38](javascript:display('ko04142')))   [ko04146 Peroxisome](https://www.genome.jp/kegg-bin/show_pathway?1531549175101874/ko04146.args) ([39](javascript:display('ko04146')))   [ko04140 Autophagy - animal](https://www.genome.jp/kegg-bin/show_pathway?1531549175101874/ko04140.args) ([40](javascript:display('ko04140')))   [ko04138 Autophagy - yeast](https://www.genome.jp/kegg-bin/show_pathway?1531549175101874/ko04138.args) ([45](javascript:display('ko04138')))   [ko04136 Autophagy - other](https://www.genome.jp/kegg-bin/show_pathway?1531549175101874/ko04136.args) ([23](javascript:display('ko04136')))   [ko04137 Mitophagy - animal](https://www.genome.jp/kegg-bin/show_pathway?1531549175101874/ko04137.args) ([14](javascript:display('ko04137')))   [ko04139 Mitophagy - yeast](https://www.genome.jp/kegg-bin/show_pathway?1531549175101874/ko04139.args) ([16](javascript:display('ko04139')))   [ko04110 Cell cycle](https://www.genome.jp/kegg-bin/show_pathway?1531549175101874/ko04110.args) ([58](javascript:display('ko04110')))   [ko04111 Cell cycle - yeast](https://www.genome.jp/kegg-bin/show_pathway?1531549175101874/ko04111.args) ([54](javascript:display('ko04111')))   [ko04112 Cell cycle - Caulobacter](https://www.genome.jp/kegg-bin/show_pathway?1531549175101874/ko04112.args) ([6](javascript:display('ko04112')))   [ko04113 Meiosis - yeast](https://www.genome.jp/kegg-bin/show_pathway?1531549175101874/ko04113.args) ([41](javascript:display('ko04113')))   [ko04114 Oocyte meiosis](https://www.genome.jp/kegg-bin/show_pathway?1531549175101874/ko04114.args) ([32](javascript:display('ko04114')))   [ko04210 Apoptosis](https://www.genome.jp/kegg-bin/show_pathway?1531549175101874/ko04210.args) ([16](javascript:display('ko04210')))   [ko04214 Apoptosis - fly](https://www.genome.jp/kegg-bin/show_pathway?1531549175101874/ko04214.args) ([12](javascript:display('ko04214')))   [ko04215 Apoptosis - multiple species](https://www.genome.jp/kegg-bin/show_pathway?1531549175101874/ko04215.args) ([3](javascript:display('ko04215')))   [ko04216 Ferroptosis](https://www.genome.jp/kegg-bin/show_pathway?1531549175101874/ko04216.args) ([7](javascript:display('ko04216')))   [ko04217 Necroptosis](https://www.genome.jp/kegg-bin/show_pathway?1531549175101874/ko04217.args) ([22](javascript:display('ko04217')))   [ko04115 p53 signaling pathway](https://www.genome.jp/kegg-bin/show_pathway?1531549175101874/ko04115.args) ([13](javascript:display('ko04115')))   [ko04218 Cellular senescence](https://www.genome.jp/kegg-bin/show_pathway?1531549175101874/ko04218.args) ([28](javascript:display('ko04218')))   [ko04510 Focal adhesion](https://www.genome.jp/kegg-bin/show_pathway?1531549175101874/ko04510.args) ([11](javascript:display('ko04510')))   [ko04520 Adherens junction](https://www.genome.jp/kegg-bin/show_pathway?1531549175101874/ko04520.args) ([6](javascript:display('ko04520')))   [ko04530 Tight junction](https://www.genome.jp/kegg-bin/show_pathway?1531549175101874/ko04530.args) ([14](javascript:display('ko04530')))   [ko04540 Gap junction](https://www.genome.jp/kegg-bin/show_pathway?1531549175101874/ko04540.args) ([7](javascript:display('ko04540')))   [ko04550 Signaling pathways regulating pluripotency of stem cells](https://www.genome.jp/kegg-bin/show_pathway?1531549175101874/ko04550.args) ([6](javascript:display('ko04550')))   [ko02024 Quorum sensing](https://www.genome.jp/kegg-bin/show_pathway?1531549175101874/ko02024.args) ([15](javascript:display('ko02024')))   [ko05111 Biofilm formation - Vibrio cholerae](https://www.genome.jp/kegg-bin/show_pathway?1531549175101874/ko05111.args) ([1](javascript:display('ko05111')))   [ko02025 Biofilm formation - Pseudomonas aeruginosa](https://www.genome.jp/kegg-bin/show_pathway?1531549175101874/ko02025.args) ([4](javascript:display('ko02025')))   [ko02026 Biofilm formation - Escherichia coli](https://www.genome.jp/kegg-bin/show_pathway?1531549175101874/ko02026.args) ([4](javascript:display('ko02026')))   [ko02030 Bacterial chemotaxis](https://www.genome.jp/kegg-bin/show_pathway?1531549175101874/ko02030.args) ([1](javascript:display('ko02030')))   [ko04810 Regulation of actin cytoskeleton](https://www.genome.jp/kegg-bin/show_pathway?1531549175101874/ko04810.args) ([20](javascript:display('ko04810')))   [ko04611 Platelet activation](https://www.genome.jp/kegg-bin/show_pathway?1531549175101874/ko04611.args) ([4](javascript:display('ko04611')))   [ko04620 Toll-like receptor signaling pathway](https://www.genome.jp/kegg-bin/show_pathway?1531549175101874/ko04620.args) ([8](javascript:display('ko04620')))   [ko04624 Toll and Imdsignaling pathway](https://www.genome.jp/kegg-bin/show_pathway?1531549175101874/ko04624.args) ([9](javascript:display('ko04624')))   [ko04621 NOD-like receptor signaling pathway](https://www.genome.jp/kegg-bin/show_pathway?1531549175101874/ko04621.args) ([19](javascript:display('ko04621')))   [ko04622 RIG-I-like receptor signaling pathway](https://www.genome.jp/kegg-bin/show_pathway?1531549175101874/ko04622.args) ([8](javascript:display('ko04622')))   [ko04623 Cytosolic DNA-sensing pathway](https://www.genome.jp/kegg-bin/show_pathway?1531549175101874/ko04623.args) ([15](javascript:display('ko04623')))   [ko04625 C-type lectin receptor signaling pathway](https://www.genome.jp/kegg-bin/show_pathway?1531549175101874/ko04625.args) ([3](javascript:display('ko04625')))   [ko04650 Natural killer cell mediated cytotoxicity](https://www.genome.jp/kegg-bin/show_pathway?1531549175101874/ko04650.args) ([6](javascript:display('ko04650')))   [ko04612 Antigen processing and presentation](https://www.genome.jp/kegg-bin/show_pathway?1531549175101874/ko04612.args) ([9](javascript:display('ko04612')))   [ko04660 T cell receptor signaling pathway](https://www.genome.jp/kegg-bin/show_pathway?1531549175101874/ko04660.args) ([7](javascript:display('ko04660')))   [ko04658 Th1 and Th2 cell differentiation](https://www.genome.jp/kegg-bin/show_pathway?1531549175101874/ko04658.args) ([2](javascript:display('ko04658')))   [ko04659 Th17 cell differentiation](https://www.genome.jp/kegg-bin/show_pathway?1531549175101874/ko04659.args) ([4](javascript:display('ko04659')))   [ko04657 IL-17 signaling pathway](https://www.genome.jp/kegg-bin/show_pathway?1531549175101874/ko04657.args) ([7](javascript:display('ko04657')))   [ko04662 B cell receptor signaling pathway](https://www.genome.jp/kegg-bin/show_pathway?1531549175101874/ko04662.args) ([6](javascript:display('ko04662')))   [ko04664 Fc epsilon RI signaling pathway](https://www.genome.jp/kegg-bin/show_pathway?1531549175101874/ko04664.args) ([6](javascript:display('ko04664')))   [ko04666 Fc gamma R-mediated phagocytosis](https://www.genome.jp/kegg-bin/show_pathway?1531549175101874/ko04666.args) ([14](javascript:display('ko04666')))   [ko04670 Leukocyte transendothelial migration](https://www.genome.jp/kegg-bin/show_pathway?1531549175101874/ko04670.args) ([2](javascript:display('ko04670')))   [ko04062 Chemokine signaling pathway](https://www.genome.jp/kegg-bin/show_pathway?1531549175101874/ko04062.args) ([7](javascript:display('ko04062')))   [ko04911 Insulin secretion](https://www.genome.jp/kegg-bin/show_pathway?1531549175101874/ko04911.args) ([3](javascript:display('ko04911')))   [ko04910 Insulin signaling pathway](https://www.genome.jp/kegg-bin/show_pathway?1531549175101874/ko04910.args) ([21](javascript:display('ko04910')))   [ko04922 Glucagon signaling pathway](https://www.genome.jp/kegg-bin/show_pathway?1531549175101874/ko04922.args) ([17](javascript:display('ko04922')))   [ko04923 Regulation of lipolysis in adipocytes](https://www.genome.jp/kegg-bin/show_pathway?1531549175101874/ko04923.args) ([3](javascript:display('ko04923')))   [ko04920 Adipocytokinesignaling pathway](https://www.genome.jp/kegg-bin/show_pathway?1531549175101874/ko04920.args) ([5](javascript:display('ko04920')))   [ko03320 PPAR signaling pathway](https://www.genome.jp/kegg-bin/show_pathway?1531549175101874/ko03320.args) ([9](javascript:display('ko03320')))   [ko04912 GnRHsignaling pathway](https://www.genome.jp/kegg-bin/show_pathway?1531549175101874/ko04912.args) ([8](javascript:display('ko04912')))   [ko04913 Ovarian steroidogenesis](https://www.genome.jp/kegg-bin/show_pathway?1531549175101874/ko04913.args) ([3](javascript:display('ko04913')))   [ko04915 Estrogensignaling pathway](https://www.genome.jp/kegg-bin/show_pathway?1531549175101874/ko04915.args) ([11](javascript:display('ko04915')))   [ko04914 Progesterone-mediated oocyte maturation](https://www.genome.jp/kegg-bin/show_pathway?1531549175101874/ko04914.args) ([23](javascript:display('ko04914')))   [ko04917 Prolactin signaling pathway](https://www.genome.jp/kegg-bin/show_pathway?1531549175101874/ko04917.args) ([5](javascript:display('ko04917')))   [ko04921 Oxytocin signaling pathway](https://www.genome.jp/kegg-bin/show_pathway?1531549175101874/ko04921.args) ([13](javascript:display('ko04921')))   [ko04926 Relaxinsignaling pathway](https://www.genome.jp/kegg-bin/show_pathway?1531549175101874/ko04926.args) ([5](javascript:display('ko04926')))   [ko04918 Thyroid hormone synthesis](https://www.genome.jp/kegg-bin/show_pathway?1531549175101874/ko04918.args) ([6](javascript:display('ko04918')))   [ko04919 Thyroid hormone signaling pathway](https://www.genome.jp/kegg-bin/show_pathway?1531549175101874/ko04919.args) ([18](javascript:display('ko04919')))   [ko04928 Parathyroid hormone synthesis, secretion and action](https://www.genome.jp/kegg-bin/show_pathway?1531549175101874/ko04928.args) ([9](javascript:display('ko04928')))   [ko04916 Melanogenesis](https://www.genome.jp/kegg-bin/show_pathway?1531549175101874/ko04916.args) ([8](javascript:display('ko04916')))   [ko04924 Renin secretion](https://www.genome.jp/kegg-bin/show_pathway?1531549175101874/ko04924.args) ([4](javascript:display('ko04924')))   [ko04614 Renin-angiotensin system](https://www.genome.jp/kegg-bin/show_pathway?1531549175101874/ko04614.args) ([4](javascript:display('ko04614')))   [ko04925 Aldosterone synthesis and secretion](https://www.genome.jp/kegg-bin/show_pathway?1531549175101874/ko04925.args) ([6](javascript:display('ko04925')))   [ko04927 Cortisol synthesis and secretion](https://www.genome.jp/kegg-bin/show_pathway?1531549175101874/ko04927.args) ([1](javascript:display('ko04927')))   [ko04260 Cardiac muscle contraction](https://www.genome.jp/kegg-bin/show_pathway?1531549175101874/ko04260.args) ([14](javascript:display('ko04260')))   [ko04261 Adrenergic signaling in cardiomyocytes](https://www.genome.jp/kegg-bin/show_pathway?1531549175101874/ko04261.args) ([11](javascript:display('ko04261')))   [ko04270 Vascular smooth muscle contraction](https://www.genome.jp/kegg-bin/show_pathway?1531549175101874/ko04270.args) ([8](javascript:display('ko04270')))   [ko04970 Salivary secretion](https://www.genome.jp/kegg-bin/show_pathway?1531549175101874/ko04970.args) ([2](javascript:display('ko04970')))   [ko04971 Gastric acid secretion](https://www.genome.jp/kegg-bin/show_pathway?1531549175101874/ko04971.args) ([3](javascript:display('ko04971')))   [ko04972 Pancreatic secretion](https://www.genome.jp/kegg-bin/show_pathway?1531549175101874/ko04972.args) ([5](javascript:display('ko04972')))   [ko04976 Bile secretion](https://www.genome.jp/kegg-bin/show_pathway?1531549175101874/ko04976.args) ([2](javascript:display('ko04976')))   [ko04973 Carbohydrate digestion and absorption](https://www.genome.jp/kegg-bin/show_pathway?1531549175101874/ko04973.args) ([4](javascript:display('ko04973')))   [ko04974 Protein digestion and absorption](https://www.genome.jp/kegg-bin/show_pathway?1531549175101874/ko04974.args) ([5](javascript:display('ko04974')))   [ko04975 Fat digestion and absorption](https://www.genome.jp/kegg-bin/show_pathway?1531549175101874/ko04975.args) ([5](javascript:display('ko04975')))   [ko04979 Cholesterol metabolism](https://www.genome.jp/kegg-bin/show_pathway?1531549175101874/ko04979.args) ([5](javascript:display('ko04979')))   [ko04977 Vitamin digestion and absorption](https://www.genome.jp/kegg-bin/show_pathway?1531549175101874/ko04977.args) ([1](javascript:display('ko04977')))   [ko04978 Mineral absorption](https://www.genome.jp/kegg-bin/show_pathway?1531549175101874/ko04978.args) ([4](javascript:display('ko04978')))   [ko04962 Vasopressin-regulated water reabsorption](https://www.genome.jp/kegg-bin/show_pathway?1531549175101874/ko04962.args) ([6](javascript:display('ko04962')))   [ko04960 Aldosterone-regulated sodium reabsorption](https://www.genome.jp/kegg-bin/show_pathway?1531549175101874/ko04960.args) ([2](javascript:display('ko04960')))   [ko04961 Endocrine and other factor-regulated calcium reabsorption](https://www.genome.jp/kegg-bin/show_pathway?1531549175101874/ko04961.args) ([7](javascript:display('ko04961')))   [ko04964 Proximal tubule bicarbonate reclamation](https://www.genome.jp/kegg-bin/show_pathway?1531549175101874/ko04964.args) ([3](javascript:display('ko04964')))   [ko04966 Collecting duct acid secretion](https://www.genome.jp/kegg-bin/show_pathway?1531549175101874/ko04966.args) ([11](javascript:display('ko04966')))   [ko04724 Glutamatergic synapse](https://www.genome.jp/kegg-bin/show_pathway?1531549175101874/ko04724.args) ([9](javascript:display('ko04724')))   [ko04727 GABAergic synapse](https://www.genome.jp/kegg-bin/show_pathway?1531549175101874/ko04727.args) ([10](javascript:display('ko04727')))   [ko04725 Cholinergic synapse](https://www.genome.jp/kegg-bin/show_pathway?1531549175101874/ko04725.args) ([5](javascript:display('ko04725')))   [ko04728 Dopaminergic synapse](https://www.genome.jp/kegg-bin/show_pathway?1531549175101874/ko04728.args) ([12](javascript:display('ko04728')))   [ko04726 Serotonergic synapse](https://www.genome.jp/kegg-bin/show_pathway?1531549175101874/ko04726.args) ([7](javascript:display('ko04726')))   [ko04720 Long-term potentiation](https://www.genome.jp/kegg-bin/show_pathway?1531549175101874/ko04720.args) ([11](javascript:display('ko04720')))   [ko04730 Long-term depression](https://www.genome.jp/kegg-bin/show_pathway?1531549175101874/ko04730.args) ([7](javascript:display('ko04730')))   [ko04723 Retrograde endocannabinoidsignaling](https://www.genome.jp/kegg-bin/show_pathway?1531549175101874/ko04723.args) ([33](javascript:display('ko04723')))   [ko04721 Synaptic vesicle cycle](https://www.genome.jp/kegg-bin/show_pathway?1531549175101874/ko04721.args) ([24](javascript:display('ko04721')))   [ko04722 Neurotrophinsignaling pathway](https://www.genome.jp/kegg-bin/show_pathway?1531549175101874/ko04722.args) ([15](javascript:display('ko04722')))   [ko04744 Phototransduction](https://www.genome.jp/kegg-bin/show_pathway?1531549175101874/ko04744.args) ([2](javascript:display('ko04744')))   [ko04745 Phototransduction - fly](https://www.genome.jp/kegg-bin/show_pathway?1531549175101874/ko04745.args) ([4](javascript:display('ko04745')))   [ko04740 Olfactory transduction](https://www.genome.jp/kegg-bin/show_pathway?1531549175101874/ko04740.args) ([5](javascript:display('ko04740')))   [ko04742 Taste transduction](https://www.genome.jp/kegg-bin/show_pathway?1531549175101874/ko04742.args) ([3](javascript:display('ko04742')))   [ko04750 Inflammatory mediator regulation of TRP channels](https://www.genome.jp/kegg-bin/show_pathway?1531549175101874/ko04750.args) ([4](javascript:display('ko04750')))   [ko04320 Dorso-ventral axis formation](https://www.genome.jp/kegg-bin/show_pathway?1531549175101874/ko04320.args) ([3](javascript:display('ko04320')))   [ko04360 Axon guidance](https://www.genome.jp/kegg-bin/show_pathway?1531549175101874/ko04360.args) ([7](javascript:display('ko04360')))   [ko04380 Osteoclast differentiation](https://www.genome.jp/kegg-bin/show_pathway?1531549175101874/ko04380.args) ([7](javascript:display('ko04380')))   [ko04211 Longevity regulating pathway](https://www.genome.jp/kegg-bin/show_pathway?1531549175101874/ko04211.args) ([13](javascript:display('ko04211')))   [ko04212 Longevity regulating pathway - worm](https://www.genome.jp/kegg-bin/show_pathway?1531549175101874/ko04212.args) ([19](javascript:display('ko04212')))   [ko04213 Longevity regulating pathway - multiple species](https://www.genome.jp/kegg-bin/show_pathway?1531549175101874/ko04213.args) ([13](javascript:display('ko04213')))   [ko04710 Circadian rhythm](https://www.genome.jp/kegg-bin/show_pathway?1531549175101874/ko04710.args) ([6](javascript:display('ko04710')))   [ko04713 Circadian entrainment](https://www.genome.jp/kegg-bin/show_pathway?1531549175101874/ko04713.args) ([5](javascript:display('ko04713')))   [ko04711 Circadian rhythm - fly](https://www.genome.jp/kegg-bin/show_pathway?1531549175101874/ko04711.args) ([2](javascript:display('ko04711')))   [ko04712 Circadian rhythm - plant](https://www.genome.jp/kegg-bin/show_pathway?1531549175101874/ko04712.args) ([21](javascript:display('ko04712')))   [ko04714 Thermogenesis](https://www.genome.jp/kegg-bin/show_pathway?1531549175101874/ko04714.args) ([86](javascript:display('ko04714')))   [ko04626 Plant-pathogen interaction](https://www.genome.jp/kegg-bin/show_pathway?1531549175101874/ko04626.args) ([27](javascript:display('ko04626')))   [ko05200 Pathways in cancer](https://www.genome.jp/kegg-bin/show_pathway?1531549175101874/ko05200.args) ([40](javascript:display('ko05200')))   [ko05230 Central carbon metabolism in cancer](https://www.genome.jp/kegg-bin/show_pathway?1531549175101874/ko05230.args) ([15](javascript:display('ko05230')))   [ko05231 Choline metabolism in cancer](https://www.genome.jp/kegg-bin/show_pathway?1531549175101874/ko05231.args) ([14](javascript:display('ko05231')))   [ko05202 Transcriptional misregulation in cancer](https://www.genome.jp/kegg-bin/show_pathway?1531549175101874/ko05202.args) ([14](javascript:display('ko05202')))   [ko05206 MicroRNAs in cancer](https://www.genome.jp/kegg-bin/show_pathway?1531549175101874/ko05206.args) ([20](javascript:display('ko05206')))   [ko05205 Proteoglycans in cancer](https://www.genome.jp/kegg-bin/show_pathway?1531549175101874/ko05205.args) ([15](javascript:display('ko05205')))   [ko05204 Chemical carcinogenesis](https://www.genome.jp/kegg-bin/show_pathway?1531549175101874/ko05204.args) ([6](javascript:display('ko05204')))   [ko05203 Viral carcinogenesis](https://www.genome.jp/kegg-bin/show_pathway?1531549175101874/ko05203.args) ([41](javascript:display('ko05203')))   [ko05210 Colorectal cancer](https://www.genome.jp/kegg-bin/show_pathway?1531549175101874/ko05210.args) ([15](javascript:display('ko05210')))   [ko05212 Pancreatic cancer](https://www.genome.jp/kegg-bin/show_pathway?1531549175101874/ko05212.args) ([12](javascript:display('ko05212')))   [ko05225 Hepatocellular carcinoma](https://www.genome.jp/kegg-bin/show_pathway?1531549175101874/ko05225.args) ([21](javascript:display('ko05225')))   [ko05226 Gastric cancer](https://www.genome.jp/kegg-bin/show_pathway?1531549175101874/ko05226.args) ([15](javascript:display('ko05226')))   [ko05214 Glioma](https://www.genome.jp/kegg-bin/show_pathway?1531549175101874/ko05214.args) ([12](javascript:display('ko05214')))   [ko05216 Thyroid cancer](https://www.genome.jp/kegg-bin/show_pathway?1531549175101874/ko05216.args) ([7](javascript:display('ko05216')))   [ko05221 Acute myeloid leukemia](https://www.genome.jp/kegg-bin/show_pathway?1531549175101874/ko05221.args) ([6](javascript:display('ko05221')))   [ko05220 Chronic myeloid leukemia](https://www.genome.jp/kegg-bin/show_pathway?1531549175101874/ko05220.args) ([9](javascript:display('ko05220')))   [ko05217 Basal cell carcinoma](https://www.genome.jp/kegg-bin/show_pathway?1531549175101874/ko05217.args) ([3](javascript:display('ko05217')))   [ko05218 Melanoma](https://www.genome.jp/kegg-bin/show_pathway?1531549175101874/ko05218.args) ([9](javascript:display('ko05218')))   [ko05211 Renal cell carcinoma](https://www.genome.jp/kegg-bin/show_pathway?1531549175101874/ko05211.args) ([10](javascript:display('ko05211')))   [ko05219 Bladder cancer](https://www.genome.jp/kegg-bin/show_pathway?1531549175101874/ko05219.args) ([6](javascript:display('ko05219')))   [ko05215 Prostate cancer](https://www.genome.jp/kegg-bin/show_pathway?1531549175101874/ko05215.args) ([14](javascript:display('ko05215')))   [ko05213 Endometrial cancer](https://www.genome.jp/kegg-bin/show_pathway?1531549175101874/ko05213.args) ([10](javascript:display('ko05213')))   [ko05224 Breast cancer](https://www.genome.jp/kegg-bin/show_pathway?1531549175101874/ko05224.args) ([13](javascript:display('ko05224')))   [ko05222 Small cell lung cancer](https://www.genome.jp/kegg-bin/show_pathway?1531549175101874/ko05222.args) ([11](javascript:display('ko05222')))   [ko05223 Non-small cell lung cancer](https://www.genome.jp/kegg-bin/show_pathway?1531549175101874/ko05223.args) ([10](javascript:display('ko05223')))   [ko05322 Systemic lupus erythematosus](https://www.genome.jp/kegg-bin/show_pathway?1531549175101874/ko05322.args) ([8](javascript:display('ko05322')))   [ko05323 Rheumatoid arthritis](https://www.genome.jp/kegg-bin/show_pathway?1531549175101874/ko05323.args) ([14](javascript:display('ko05323')))   [ko05340 Primary immunodeficiency](https://www.genome.jp/kegg-bin/show_pathway?1531549175101874/ko05340.args) ([3](javascript:display('ko05340')))   [ko05010 Alzheimer disease](https://www.genome.jp/kegg-bin/show_pathway?1531549175101874/ko05010.args) ([59](javascript:display('ko05010')))   [ko05012 Parkinson disease](https://www.genome.jp/kegg-bin/show_pathway?1531549175101874/ko05012.args) ([58](javascript:display('ko05012')))   [ko05014 Amyotrophic lateral sclerosis (ALS)](https://www.genome.jp/kegg-bin/show_pathway?1531549175101874/ko05014.args) ([7](javascript:display('ko05014')))   [ko05016 Huntington disease](https://www.genome.jp/kegg-bin/show_pathway?1531549175101874/ko05016.args) ([74](javascript:display('ko05016')))   [ko05020 Prion diseases](https://www.genome.jp/kegg-bin/show_pathway?1531549175101874/ko05020.args) ([6](javascript:display('ko05020')))   [ko05030 Cocaine addiction](https://www.genome.jp/kegg-bin/show_pathway?1531549175101874/ko05030.args) ([1](javascript:display('ko05030')))   [ko05031 Amphetamine addiction](https://www.genome.jp/kegg-bin/show_pathway?1531549175101874/ko05031.args) ([6](javascript:display('ko05031')))   [ko05032 Morphine addiction](https://www.genome.jp/kegg-bin/show_pathway?1531549175101874/ko05032.args) ([3](javascript:display('ko05032')))   [ko05033 Nicotine addiction](https://www.genome.jp/kegg-bin/show_pathway?1531549175101874/ko05033.args) ([1](javascript:display('ko05033')))   [ko05034 Alcoholism](https://www.genome.jp/kegg-bin/show_pathway?1531549175101874/ko05034.args) ([15](javascript:display('ko05034')))   [ko05418 Fluid shear stress and atherosclerosis](https://www.genome.jp/kegg-bin/show_pathway?1531549175101874/ko05418.args) ([14](javascript:display('ko05418')))   [ko05410 Hypertrophic cardiomyopathy (HCM)](https://www.genome.jp/kegg-bin/show_pathway?1531549175101874/ko05410.args) ([2](javascript:display('ko05410')))   [ko05416 Viral myocarditis](https://www.genome.jp/kegg-bin/show_pathway?1531549175101874/ko05416.args) ([4](javascript:display('ko05416')))   [ko04930 Type II diabetes mellitus](https://www.genome.jp/kegg-bin/show_pathway?1531549175101874/ko04930.args) ([4](javascript:display('ko04930')))   [ko04940 Type I diabetes mellitus](https://www.genome.jp/kegg-bin/show_pathway?1531549175101874/ko04940.args) ([2](javascript:display('ko04940')))   [ko04932 Non-alcoholic fatty liver disease (NAFLD)](https://www.genome.jp/kegg-bin/show_pathway?1531549175101874/ko04932.args) ([45](javascript:display('ko04932')))   [ko04931 Insulin resistance](https://www.genome.jp/kegg-bin/show_pathway?1531549175101874/ko04931.args) ([13](javascript:display('ko04931')))   [ko04933 AGE-RAGE signaling pathway in diabetic complications](https://www.genome.jp/kegg-bin/show_pathway?1531549175101874/ko04933.args) ([5](javascript:display('ko04933')))   [ko04934 Cushing syndrome](https://www.genome.jp/kegg-bin/show_pathway?1531549175101874/ko04934.args) ([14](javascript:display('ko04934')))   [ko05110 Vibrio cholerae infection](https://www.genome.jp/kegg-bin/show_pathway?1531549175101874/ko05110.args) ([19](javascript:display('ko05110')))   [ko05120 Epithelial cell signaling in Helicobacter pylori infection](https://www.genome.jp/kegg-bin/show_pathway?1531549175101874/ko05120.args) ([15](javascript:display('ko05120')))   [ko05130 Pathogenic Escherichia coli infection](https://www.genome.jp/kegg-bin/show_pathway?1531549175101874/ko05130.args) ([8](javascript:display('ko05130')))   [ko05132 Salmonella infection](https://www.genome.jp/kegg-bin/show_pathway?1531549175101874/ko05132.args) ([10](javascript:display('ko05132')))   [ko05131 Shigellosis](https://www.genome.jp/kegg-bin/show_pathway?1531549175101874/ko05131.args) ([12](javascript:display('ko05131')))   [ko05133 Pertussis](https://www.genome.jp/kegg-bin/show_pathway?1531549175101874/ko05133.args) ([5](javascript:display('ko05133')))   [ko05134 Legionellosis](https://www.genome.jp/kegg-bin/show_pathway?1531549175101874/ko05134.args) ([12](javascript:display('ko05134')))   [ko05152 Tuberculosis](https://www.genome.jp/kegg-bin/show_pathway?1531549175101874/ko05152.args) ([23](javascript:display('ko05152')))   [ko05100 Bacterial invasion of epithelial cells](https://www.genome.jp/kegg-bin/show_pathway?1531549175101874/ko05100.args) ([10](javascript:display('ko05100')))   [ko05166 HTLV-I infection](https://www.genome.jp/kegg-bin/show_pathway?1531549175101874/ko05166.args) ([45](javascript:display('ko05166')))   [ko05162 Measles](https://www.genome.jp/kegg-bin/show_pathway?1531549175101874/ko05162.args) ([15](javascript:display('ko05162')))   [ko05164 Influenza A](https://www.genome.jp/kegg-bin/show_pathway?1531549175101874/ko05164.args) ([21](javascript:display('ko05164')))   [ko05161 Hepatitis B](https://www.genome.jp/kegg-bin/show_pathway?1531549175101874/ko05161.args) ([15](javascript:display('ko05161')))   [ko05160 Hepatitis C](https://www.genome.jp/kegg-bin/show_pathway?1531549175101874/ko05160.args) ([11](javascript:display('ko05160')))   [ko05168 Herpes simplex infection](https://www.genome.jp/kegg-bin/show_pathway?1531549175101874/ko05168.args) ([32](javascript:display('ko05168')))   [ko05163 Human cytomegalovirus infection](https://www.genome.jp/kegg-bin/show_pathway?1531549175101874/ko05163.args) ([15](javascript:display('ko05163')))   [ko05167 Kaposi sarcoma-associated herpesvirus infection](https://www.genome.jp/kegg-bin/show_pathway?1531549175101874/ko05167.args) ([17](javascript:display('ko05167')))   [ko05169 Epstein-Barr virus infection](https://www.genome.jp/kegg-bin/show_pathway?1531549175101874/ko05169.args) ([66](javascript:display('ko05169')))   [ko05165 Human papillomavirus infection](https://www.genome.jp/kegg-bin/show_pathway?1531549175101874/ko05165.args) ([46](javascript:display('ko05165')))   [ko05146 Amoebiasis](https://www.genome.jp/kegg-bin/show_pathway?1531549175101874/ko05146.args) ([5](javascript:display('ko05146')))   [ko05144 Malaria](https://www.genome.jp/kegg-bin/show_pathway?1531549175101874/ko05144.args) ([1](javascript:display('ko05144')))   [ko05145 Toxoplasmosis](https://www.genome.jp/kegg-bin/show_pathway?1531549175101874/ko05145.args) ([10](javascript:display('ko05145')))   [ko05140 Leishmaniasis](https://www.genome.jp/kegg-bin/show_pathway?1531549175101874/ko05140.args) ([5](javascript:display('ko05140')))   [ko05142 Chagas disease (American trypanosomiasis)](https://www.genome.jp/kegg-bin/show_pathway?1531549175101874/ko05142.args) ([10](javascript:display('ko05142')))   [ko05143 African trypanosomiasis](https://www.genome.jp/kegg-bin/show_pathway?1531549175101874/ko05143.args) ([5](javascript:display('ko05143')))   [ko01501 beta-Lactam resistance](https://www.genome.jp/kegg-bin/show_pathway?1531549175101874/ko01501.args) ([1](javascript:display('ko01501')))   [ko01502 Vancomycin resistance](https://www.genome.jp/kegg-bin/show_pathway?1531549175101874/ko01502.args) ([2](javascript:display('ko01502')))   [ko01503 Cationic antimicrobial peptide (CAMP) resistance](https://www.genome.jp/kegg-bin/show_pathway?1531549175101874/ko01503.args) ([2](javascript:display('ko01503')))   [ko01521 EGFR tyrosine kinase inhibitor resistance](https://www.genome.jp/kegg-bin/show_pathway?1531549175101874/ko01521.args) ([10](javascript:display('ko01521')))   [ko01524 Platinum drug resistance](https://www.genome.jp/kegg-bin/show_pathway?1531549175101874/ko01524.args) ([16](javascript:display('ko01524')))   [ko01523 Antifolate resistance](https://www.genome.jp/kegg-bin/show_pathway?1531549175101874/ko01523.args) ([6](javascript:display('ko01523')))   [ko01522 Endocrine resistance](https://www.genome.jp/kegg-bin/show_pathway?1531549175101874/ko01522.args) ([10](javascript:display('ko01522'))) |
| --- |
|  |
|  |
|  |
|  |
|  |
|  |
|  |
