# Supplementary material for: Identification and characterization of SSR, SNP and InDel molecular markers from RNA-Seq data of guar (Cyamopsis tetragonoloba, L. Taub.) roots
Source: BMC Genomics. 2018 Dec 20;19:951. doi: 10.1186/s12864-018-5205-9 (PMC6302463; doi:10.1186/s12864-018-5205-9)
Supplement: Supplementary file 19 — Table S17. List of the primers used to study the SNPs present in root development genes of guar. (DOCX 12 kb) [file 12864_2018_5205_MOESM19_ESM.docx]

Table S17. List of the primers used to study the SNPs present in root development genes of guar.

| S.No | SNP Name | Forward Primer | Reverse Primer |
| --- | --- | --- | --- |
| 1 | OT3537 | TGTTCAGTTTGGAAC | CCTTGGCCTGGAAAT |
| 2 | OT2789 | TGTTCAGTTTGGAAC | CCTTGGCCTGGAAAT |
| 3 | OT2790 | AGGTAGCAGCCGCTT | CCTTGGCCTGGAAAT |
| 4 | OT2791 | CAACCTACATCTGCT | CCTTGGCCTGGAAAT |
| 5 | OT2792 | CAACCTACATCTGCT | CCTTGGCCTGGAAAT |
| 6 | OT2793 | ATTGGCATATCGATC | CCTTGGCCTGGAAAT |
| 7 | OT2794 | CGTCCTCAAGGGCTT | CCTTCTTTCTCTTCT |
| 8 | OT4846 | ACCAACAAGCCTACT | AAGTGCAACTTTCCT |
| 9 | OT1382 | GCAGAAATCCCTACA | GATGAGACTTCAAGA |
| 10 | OT3553 | TCTGCGACTCAAAAG | ACTTGAATCTTGCTT |
| 11 | OT3554 | GGTACAGTTACTTAT | CCCTTTCACTCTTTG |
| 12 | OT3555 | GGTACAGTTACTTAT | TCTTTGCTGTGTTTG |
| 13 | OT3861 | CTTAAAAGGATGGAG | TTCTGTAGAGGATTT |
| 14 | OT3862 | ACCAAGAGACAACCC | GTAGTCATGTTCAAA |
| 15 | OT3863 | TGACTGGTTTAATTT | GTTGTTGCAACTCCA |
| 16 | OT4656 | ATGTGGATTGGTGGA | ACCATTTAATTTCTT |
