# Supplementary material for: Identification and characterization of SSR, SNP and InDel molecular markers from RNA-Seq data of guar (Cyamopsis tetragonoloba, L. Taub.) roots
Source: BMC Genomics. 2018 Dec 20;19:951. doi: 10.1186/s12864-018-5205-9 (PMC6302463; doi:10.1186/s12864-018-5205-9)
Supplement: Supplementary file 20 — Table S18. List of the primers used to study the SNPs located in guar unigenes involved in biotic and abiotic stress responses. (DOCX 13 kb) [file 12864_2018_5205_MOESM20_ESM.docx]

**Table S18. List of the primers used to study the SNPs located in guar unigenes involved in biotic and abiotic stress responses.**

| S.No | SNP Name | Forward Primer | Reverse Primer |
| --- | --- | --- | --- |
| 1 | OT769 | ATAACTCACGGACTT | CTTGCTTTCCGTGAA |
| 2 | OT1113 | TCTTCCTCTATGCTT | AGATTGCTGAATTGA |
| 3 | OT1114 | AAGAACCTTTGGACA | AGATTGCTGAATTGA |
| 4 | OT1115 | TCTGGAAGTACAGCA | AGATTGCTGAATTGA |
| 5 | OT1116 | TGTGAGAAGGAACCG | CTACACTTGCTAAGT |
| 6 | OT1161 | CAAGTGCAGTGAGGT | ACACTCTACATTGGC |
| 7 | OT1162 | TTTGTAAGCGATGTG | ACACTCTACATTGGC |
| 8 | OT1163 | GCAAATTGTAAAGGT | TGGATGGCAGAAGGC |
| 9 | OT1431 | AGATGCTGTGGCCGA | AACCCACCCTCACTC |
| 10 | OT1432 | CAGAGATCCACATGT | AACCCACCCTCACTC |
| 11 | OT1433 | ATGGCTTGATTCTGT | AACCCACCCTCACTC |
| 12 | OT1434 | ATGGCTTGATTCTGT | AACCCACCCTCACTC |
| 13 | OT1435 | ATGGCTTGATTCTGT | AACCCACCCTCACTC |
| 14 | OT1436 | TCTTGGATGAATTAC | AAGGCTAGAGGCAAT |
| 15 | OT1437 | TTCAACCGTGCGTGC | AAGGCTAGAGGCAAT |
| 16 | OT1881 | ATAAATTCTTCCATT | TTGGGCGATGTCTTC |
| 17 | OT1916 | GAAGACCTAAAAGTG | GAAATGGCAGATCTC |
| 18 | OT2923 | GCAAAGCAAATTGGC | ACCCTTGAAATTGGA |
| 19 | OT2924 | GCAAAGCAAATTGGC | AAAGTTGAGCATGAG |
| 20 | OT3720 | TTGAGTAGCAAGACC | TGAATTTTGTCCTTT |
| 21 | OT67 | TCTTATAACTTCGAT | TGTGATGTTAATGAA |
| 22 | OT458 | TGGAAGCACCAGATT | TCCTCGGCGTCTCCA |
| 23 | OT543 | TGATGACGAAAATGC | AATTTCAATCACACG |
| 24 | OT3934 | TACAAGCAAAGTACA | AATTCACTGCCTTGC |
| 25 | OT3935 | TCTTGGAATCTGCAT | AATTCACTGCCTTGC |
| 26 | OT1691 | TTGGGAGAAGAAGGC | AGCAGCAGACGGCGT |
| 27 | OT1738 | TCTAATGTTCCACCA | TGCTCGAGTCATCCA |

*.*
